# The Disintegrin and Metalloprotease ADAM12 Is Associated with TGF-β-Induced Epithelial to Mesenchymal Transition

**DOI:** 10.1371/journal.pone.0139179

**Published:** 2015-09-25

**Authors:** Michaël Ruff, Anthony Leyme, Fabienne Le Cann, Dominique Bonnier, Jacques Le Seyec, Franck Chesnel, Laurent Fattet, Ruth Rimokh, Georges Baffet, Nathalie Théret

**Affiliations:** 1 INSERM U1085, IRSET, Université de Rennes 1, Rennes, France; 2 CNRS UMR6290 IGDR, Université de Rennes 1, Rennes, France; 3 INSERM U1052, Centre de Recherche en Cancérologie de Lyon, Lyon, France; National Cancer Center, JAPAN

## Abstract

The increased expression of the Disintegrin and Metalloprotease ADAM12 has been associated with human cancers, however its role remain unclear. We have previously reported that ADAM12 expression is induced by the transforming growth factor, TGF-β and promotes TGF-β-dependent signaling through interaction with the type II receptor of TGF-β. Here we explore the implication of ADAM12 in TGF-β-mediated epithelial to mesenchymal transition (EMT), a key process in cancer progression. We show that ADAM12 expression is correlated with EMT markers in human breast cancer cell lines and biopsies. Using a non-malignant breast epithelial cell line (MCF10A), we demonstrate that TGF-β-induced EMT increases expression of the membrane-anchored ADAM12L long form. Importantly, ADAM12L overexpression in MCF10A is sufficient to induce loss of cell-cell contact, reorganization of actin cytoskeleton, up-regulation of EMT markers and chemoresistance. These effects are independent of the proteolytic activity but require the cytoplasmic tail and are specific of ADAM12L since overexpression of ADAM12S failed to induce similar changes. We further demonstrate that ADAM12L-dependent EMT is associated with increased phosphorylation of Smad3, Akt and ERK proteins. Conversely, inhibition of TGF-β receptors or ERK activities reverses ADAM12L-induced mesenchymal phenotype. Together our data demonstrate that ADAM12L is associated with EMT and contributes to TGF-β-dependent EMT by favoring both Smad-dependent and Smad-independent pathways.

## Introduction

ADAM12 is a member of the ADAM (a metalloprotease and disintegrin) protein family, a class of cell surface glycoproteins whose functions have been implicated with cell adhesion, migration, proteolysis and signaling [1]. During the last decade, ADAM12 emerged as the most strongly functional ADAM in human tumor development. Up-regulation of ADAM12 has been described in numerous cancers, including breast [2–5], colon [2], hepatocellular carcinomas [6], glioblastomas [7], stomach [2,8], oral cavity [9], bladder [10], lung [11,12] and giant cell tumors of bone [13]. ADAM12 has been shown to regulate tumor progression in mouse models either by increasing tumor cell resistance to apoptosis [3], by providing stromal support [14] or by inducing cell proliferation [15]. In addition genome-wide analyses of human breast and colorectal cancers identified ADAM12 as a new candidate cancer gene [16]. As a whole, ADAM12 is now considered as a negative prognosis marker for human bladder [10,17] and breast cancers [18,19] and is suggested to be an important player in tumor-stromal crosstalk that supports tumor progression [20].

At the molecular level, human ADAM12 exists as two alternatively splicing forms. The long transmembrane form (ADAM12L) is composed of pro-, metalloprotease, disintegrin, cysteine-rich, and transmembrane domains and a cytoplasmic tail. The short secreted form (ADAM12S) lacks the C-terminal transmembrane and cytoplasmic domains. Recent finding enlightened the differential role of the long and short forms of ADAM12 leading to the hypothesis that ADAM12L might be involved in the early-stage of breast cancer and ADAM12S might be rather implicated in the migration and invasion of cancer cell [15]. While the role of ADAM12S in cancer has been associated with its proteolytic activity, ADAM12L has been demonstrated to exhibit oncogenic properties through protease-dependent and -independent activities [21]. ADAM12L interacts with 14 proteins involved in signaling pathways and we recently demonstrated that they form together a highly connected protein network [22].

Importantly, most of these components are implicated in TGF-β signaling pathways which play a pivotal role in epithelial-mesenchymal transition (EMT), cell proliferation and metastasis [23]. TGF-β signals through a heteromeric complex of two types of transmembrane serine/threonine kinases, the type I (TβRI) and type II (TβRII) receptors. TGF-β binding to TβRII induces the recruitment and phosphorylation of TβRI which transduces signals to downstream intracellular substrates, the Smad proteins. Alternatively, non-canonical Smad pathways initiated by TGF-β receptors include Ras/MAP-kinase pathway, Jun N-terminal kinase (JNK) and p38 MAP-kinase pathways, Rho-like GTPase signaling pathways, and phosphatidylinositol-3-kinase/AKT pathway. We have previously identified ADAM12 as an interacting partner of TβRII which increases phosphorylation of the signaling Smad proteins and up-regulates TGF-β transcriptional activity and receptor trafficking [24,25]. We have also identified two other ADAM12-binding proteins that include RACK1 [26] a receptor for protein kinase C and partner of Smad3 [27] and the integrin linked kinase ILK [22] a key player in TGF-β-dependent EMT [28]. Additionally, ADAM12 has been shown to bind beta 1 integrin (ITGB1), the regulatory subunit of phosphoinositide-3-kinase p85α (PI3KR1), the adaptor protein Grb2 and the protein kinase C PKC-delta [21] which have been involved in TGF-β-dependent EMT [29]. Similarly, ADAM12 interacts with the proto-oncogene Src [30] and beta 3 integrin ITGB3 [31] which are required for TβRII phosphorylation and EMT [32].

In this study we investigated the functional association of ADAM12 with TGF-β-dependent EMT in cancer. We show that ADAM12 expression is correlated with expression of EMT markers in human breast tumor samples and breast cancer cell lines. Using the non-malignant breast epithelial cell line MCF10A, we demonstrate that TGF-β treatment induces ADAM12L expression during the course of TGF-β-dependent EMT. Importantly forced expression of ADAM12L in MCF10A cells induces loss of polarity, reorganization of actin cytoskeleton, up-regulation of EMT markers and chemoresistance. These effects are independent of its proteolytic activity and are associated with the activation of ERK-dependent pathways.

## Material and Methods

### Tissue Samples

Human tissue samples were obtained from the Biological Resource Center of Centre Léon Bérard (http://www.centreleonberard.fr/) after approval by the Comité de Protection des Personnes Lyon-Est and by the institutional review board and ethics committee of Centre Léon Bérard (French agreement number: DC-2008-99). Patient gave written informed consent after acquisition of information from the physicians. Tissue specimens were collected before any therapy from 89 patients suffering from breast cancer diagnosed between 1993 and 2001. The mean patient age at surgery was 51 + 11years (range, 27–83 years). The mean size of tumors was 3.9 + 3.1 cm and forty-four percent of the patients showed metastasis at the time of diagnosis.

### Cell Culture and Treatment

The human breast cancer cell lines (S1 Table) were generously provided by Dr. R. Rimokh (CRCL, Lyon) and grown in different cell culture media as described [33]. The mammary epithelial cell line MCF10A was obtained from American Type Culture Collection and MCF10A cells infected with LXSN-K-RasV12 or an empty vector [34] were provided by Dr BH Park (Baltimore, MD, USA). Primary human mammary epithelial cells (HMEC) infected with a retrovirus carrying hTERT, H-Ras-V12 Ras and SV40 large T antigen were provided by Dr RA Weinberg (Cambridge, MA, USA) [35]. HMECs immortalized by hTERT and Ras were designated HMEC-TR. HMECs immortalized by SV40 large T, hTERT and Ras were designated HMEC-LTR. The human mammary epithelial cells (MCF10A) were cultured in (1:1) DMEM:F12 medium supplemented with 5% horse serum, 100 ng/ml cholera toxin, 20 ng/ml EGF, 500 ng/ml hydrocortisone, and 10 μg/ml insulin. Epithelial mesenchymal cell transition was induced by treatment with recombinant TGF-β1 (Peprotech, France) at 5 ng/ml. The expression vectors for GFP-ADAM12L, ADAM12S and the protease inactive mutant ADAM12L-E351Q, were a gift from Dr. U. Wewer (Biotech Research & Innovation Centre (BRIC), University of Copenhagen). To generate lentiviral constructs, full length GFP-ADAM12L was subcloned into the pLVX-Puro vector (Clontech) and ADAM12L-E351Q and ADAM12S were subcloned into the pLVX-AcGFP1-N1 vector (Clontech). To produce viral particles, HEK293T cells were transfected with lentiviral constructs and the packaging vectors pMD2.G and psPAX2 using calcium phosphate transfection. At 48 hours after transfection, supernatant containing lentiviruses were collected and centrifugated at 130000g for 2h at 4°C on a 20% sucrose cushion to concentrate viruses. MCF10A cells were incubated with lentiviral particles for 48h and next selected and maintained in medium supplemented with 2μg/ml puromycin. Cytofluorimetric methods were used to enrich in positive cells before all experiments. For ADAM12 knockdown, MCF10A cells were incubated with GIPZ Lentiviral shADAM12 Transduction Particles (VGH5518 from Fisher Scientific, France) or with non-targeting shRNA control particles according to the manufacturer’s instructions. When indicated, cells were treated with TβR1inhibitor, SB431542 (10μM), ERK/MEK inhibitor U0126 (10 μM) and PI3K inhibitor, Wortmannin (10 μM).

### Relative Quantification of mRNA

Real-time quantitative PCR (RT-qPCR) was performed using the fluorescent dye SYBR Green methodology with the SybrTM Green I qPCRTM Core Kit (Eurogentec, Seraing, Belgium) and the ABI Prism 7700 (Perkin-Elmer, Foster city, CA, USA. The sequences of primer pairs are described in Table 1.

**Table 1:** List of oligonucleotide primer pairs used in real time RT-PCR.

Name	Sense	Anti-sense
**ADAM12L**	CAC CAT TGA AAA ACT AAG GTG TGT	GAG CCT GAC AGG GTT GGA AG
**ADAM12S**	CTG GGC ACC TCC CTT CTG	TGC TTC TGC TTG CCG GA
**Vimentin**	CCA AAC TTT TCC TCC CTG AAC C	GTG ATG CTG AGA AGT TTC GTT GA
**N-cadherin**	GTG CAT GAA GGA CAG CCT	ATG CCA TCT TCA TCC ACC TT
**E-cadherin**	GTC ATC CAA CGG GAA TGC A	TGA TCG GTT ACC GTG ATC AAA A
**TGF-β**	TGC GCT TGA GAT CTT CAA ACA	GGG CTA GTC GCA CAG ACC TC
**Twist**	GCA AGA TTC AGA CCC TCA AGC	CTC CAT CCT CCA GAC CGA GA
**Fibronectin**	GAA GAG CGA GCC CCT GAT	GGG GTC TTT TGA ACT GTG GA
**β-catenin**	AAA ATG GCA GTG CGT TTA G	TTT GAA GGC AGT CTG TCG TA
**18S**	CGC CGC TAG AGG TGA AAT TC	TTG GCA AAT GCT TTC GCT C

### Western Blotting

Cells lysates were subjected to SDS-polyacrylamide gel electrophoresis and transferred to nitrocellulose membranes (GE Healthcare, UK). The membranes were incubated for 1 hour in Tris- buffered saline containing 0.1% Tween 20 and 5% non-fat dry milk and incubated overnight with primary antibodies. The bound antibodies were visualized using horseradish peroxidase-conjugated antibodies against rabbit or mouse IgGs (DAKO, Les Ulis, France) using an enhanced chemiluminescence system (Millipore, Billerica, MA, USA). Antibodies were; ADAM12 polyclonal antibody from Sigma-Aldrich. Anti-α-phospho-p38 (Thr180/Tyr182), anti-p38 MAPK, anti-Smad2 (86F7); anti-phospho-Smad2 (Ser465/467), anti-Smad3, anti-phospho-Smad3 (Ser423/425), anti-phospho-Akt (Ser473) (D9E), anti-Akt (C67E7), anti-phospho-ERK1/2 (T202/Y204) (E10), anti-ERK1/2 (137F5), anti-phospho-JNK (Thr183/Thr185) (G9), E-cadherin (24E10), N-cadherin, anti-cleaved caspase 3 (Asp175) (5A1E) and anti-Bid were from Cell Signaling (Saint Quentin en Yvelines, France). Anti-actin and anti-JNK were from Santa Cruz Biotechnology (CA, USA). Anti-vimentin (V9) was from Dako. ADAM12 enrichment from MCF10A cell lysates was performed using affinity chromatography on a HiTrap Con A column. Briefly protein extracts were homogenized in binding buffer (20 mM Tris-HCl, pH 7.5, 500 mM NaCl, 0.05% Chaps). After several washes with the binding buffer, proteins were eluted with 20 mM Tris-HCl (pH 7.5), 500 mM NaCl, 0.2% Chaps and 0.5 M methyl a-D-mannopyranoside. Densitometry analyses were performed with ImageJ software.

### Immunostaining and Imaging

To detect actin, E-cadherin and vimentin in MCF10A, cells were fixed with 4% paraformaldehyde for 15min and permeabilized with 0.1% Triton X-100 in PBS before incubation with rabbit E-cadherin antibody (Cell Signaling) followed by alexa 555-conjugated anti-rabbit (Cell Signaling), vimentin antibody (Dako) followed by alexa 555-conjugated anti-mouse (Cell Signaling) or rhodamine-phalloïdin (Interchim, Montluçon, France). The slides were washed, mounted and viewed using an automated LEICA DMRXA2 microscope.

### Cell Proliferation

MTT assay was used to measure cell proliferation and survival. Briefly, cell numbers were investigated by incubating cells with MTT for 2 h. The resultant formazan was dissolved in DMSO, and absorbance was measured at 550 nm on Spectramax microplate reader (Molecular Devices, Wokingham, UK).

### Migration Assay

For Wound Healing assays, the confluent monolayer was scratched using a pipette tip and incubated with 2.5 μg/ml mitomycin without serum and images were captured using an automated LEICA DMRXA2 microscope at the indicated times. For Boyden chambers assays (Millipore, Molsheim, France), epithelial cells (3 × 10^5^ cells per chamber) were added to the top chamber in low serum (1%)-containing medium. The bottom chamber was filled with low serum (1%)-containing medium. Cells were cultured for 24 h at 37°C. To quantify migration, cells were mechanically removed from the top side of the membrane using a cotton-tipped swab, and migrating cells from the reverse side were fixed with methanol and stained with Giemsa. For each experiment, five representative pictures were taken for each insert, then cells were lysed and absorbance at 560 nm correlated to the amount of Giemsa stain was measured.

### Soft Agarose Colony Formation Assay

Anchorage-independent cell growth was determined by analyzing colony formation of cells in soft agar. Cells (1 × 10^4^) were resuspended in 1 ml of top agar (MCF10A media containing 0.35% Noble agar (USB Corp., Cleveland, OH) warmed to 40°C). The cell suspension was layered onto 1 ml of set bottom agar (MCF10A media containing 0.5% Noble agar) in a 6-well plate. One milliliter of medium was added on the top agar and changed once per week to compensate for evaporation. Colonies greater than 100 cells were scored after 4 weeks.

### Viability Assay

For cytotoxicity assays, cells (2.5×10^4^) were seeded in 96-well plates. Cells were treated with cisplatin or FasL for the indicated time and cell viability was determined by the MTT assay. Absorbance was measured at 550nm on Spectramax microplate reader (Molecular Devices, Wokingham, UK), and percentage of viability was calculated as the absorbance ratio of treated to untreated cells.

### Caspase-3/7 Activity

For cisplatin and FasL-induced toxicity experiments, caspase-3/7 activity was measured using ‘SensoLyte Homogeneous AMC Caspase-3/7 Assay’ kit (Anaspec, Le-Perray-en-Yvelines, France) following manufacturer’s instructions. Briefly, cells were incubated with the caspases-3/7 substrate Asp-Glu-Val-Asp-7-amino-4-methylcoumarin (DEVD-AMC) for 1h at 37°C and cleavage of the substrate AMC by caspases was measured by spectrofluorometry (Molecular Devices) at 380/440nm (ex/em). Results were expressed as Vmax.

### Bioinformatic Tool and Database

Gene array data were extracted from the genomics data repository, Gene Expression Omnibus (http://www.ncbi.nlm.nih.gov/geo/). Text mining analysis was performed using CoPub tool (http://services.nbic.nl/copub5).

## Results

### ADAM12 Is Associated with the Expression of Mesenchymal Markers in Breast Cancer Cell Lines and Human Breast Tumors

**Fig 1:**
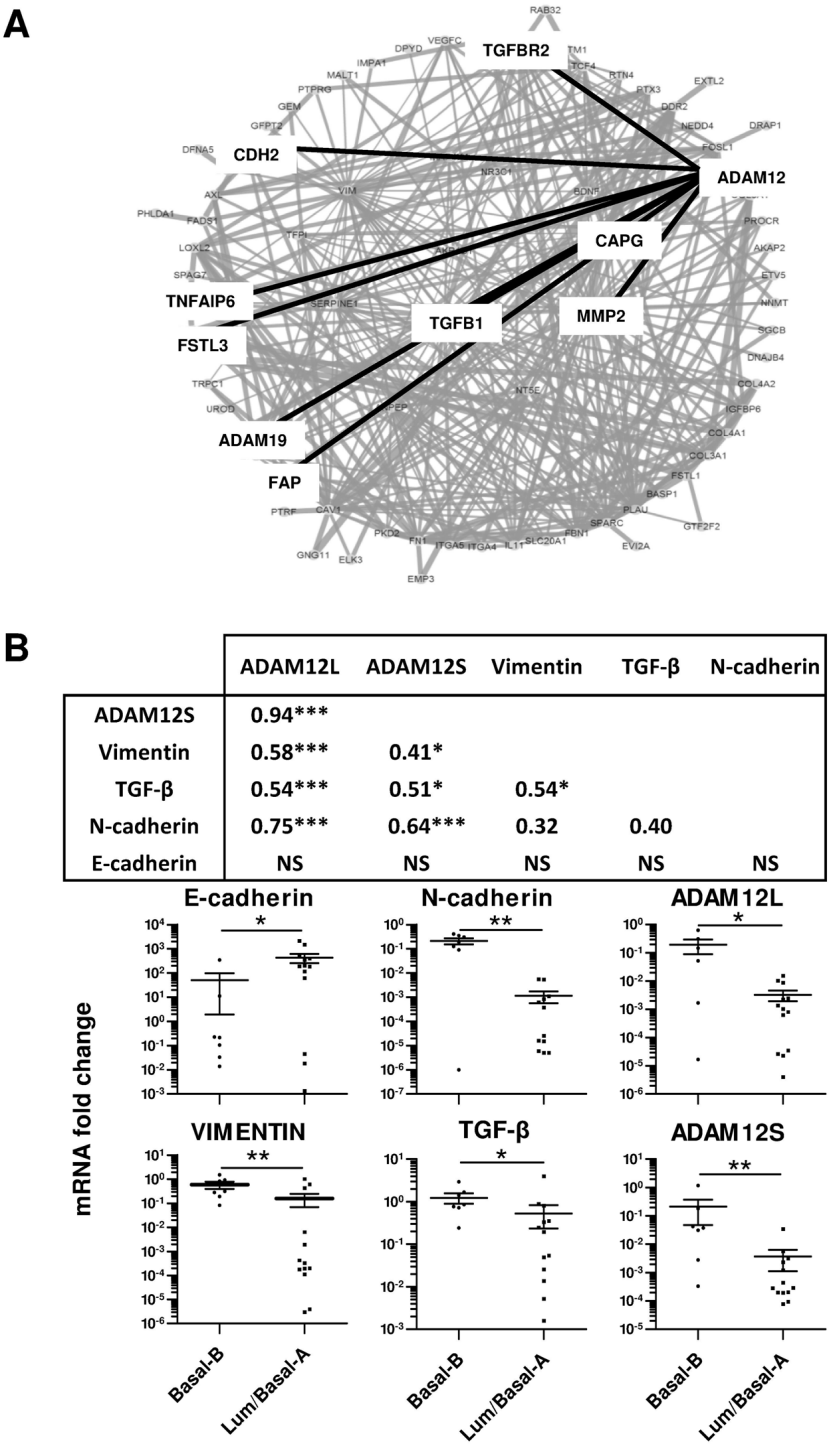
ADAM12 is associated with mesenchymal-like phenotype of Basal-B cell lines. Based on gene expression data of breast cell lines (n = 51) from Neve et al.[36] and Kao et al.[37], statistical analyses identified 635 overexpressed genes in Basal-B cell lines compared with Luminal and Basal-A cells (Detailed in S2 Table). (A) Text mining analysis of the 100 most statistically significant upregulated genes in Basal-B cell lines using CoPub tool. Nodes represent genes and edges represent co-citation in abstract. Black lines indicate ADAM12 connections. (B) Spearman correlation analyses of steady-state ADAM12L, ADAM12S, vimentin, E-cadherin and N-cadherin mRNA levels (Top). Comparative distribution of mRNA levels of ADAM12, vimentin, E-cadherin, N-cadherin and TGF-β between Lum/Basal-A and Basal B cell lines (Bottom). (***,p<0.001; **,p<0.01; *, p<0.005).

To evaluate the putative implication of ADAM12 in EMT, we first performed *in silico* analyses using transcriptional data previously reported for breast cancer cell lines [36,37]. Cell lines have been shown to cluster into basal-like and luminal expression subsets and the basal-like cell lines resolved into two distinctive clusters, Basal A and Basal B. [38]. Because Basal B cells have a mesenchymal-like appearance while Basal A cells exhibit luminal-like morphology, we searched for genes differentially expressed between Basal B cell lines and the more differentiated Luminal and Basal A cell lines. For that purpose, microarray data were extracted from Gene Expression Omnibus (GEO) database repository and we performed new analyses using SAM (Significance Analysis of Microarrays) tool. As detailed in S2 Table, we identified 665 genes overexpressed in Basal B cells including ADAM12 and EMT-associated genes such as CDH2, SNAI2 and VIM. To further characterize the relationships between these genes, we performed a text mining analysis of the 100 most statistically significant genes to build clusters of co-cited genes. As shown in Fig 1A, ADAM12 is linked with several members of TGF-β signaling such as TGFB1 itself, the receptor TGFBR2 and the antagonizing protein FSTL3 but also with the metalloproteases MMP2 and ADAM19, the N-cadherin (CDH2), the fibroblast activation protein FAP, the actin regulatory protein CAPG and a member of the hyaluronan-binding protein family, TNFAIP6.

To confirm these data, we performed new RT-PCR experiments on an independent set of 20 breast cancer cell lines. We observed again a strong correlation between expression of ADAM12L and ADAM12S with expression of N-cadherin (R = 0.75 and R = 0.64, p<0.001) and vimentin (R = 0.58 and R = 0.41, p<0.001) but not with that of E-cadherin (Fig 1B). In agreement with the meta-analyses, we found increased expression of ADAM12L and ADAM12S in Basal B cell lines compared with Luminal/BasalA cell lines. To further validate the association of ADAM12 expression with EMT, we used primary human mammary epithelial cells (HMECs), immortalized by hTERT and Ras (HMEC-TR) or by SV40 large T, hTERT and Ras (HMEC-LTR) that we treated with TGF-β to induce EMT as previously described [39]. We showed again that ADAM12 expression is highly induced in TGF-β-treated cells with mesenchymal phenotype and this expression is correlated with vimentin and N-cadherin expression (S1 Fig).

**Fig 2:**
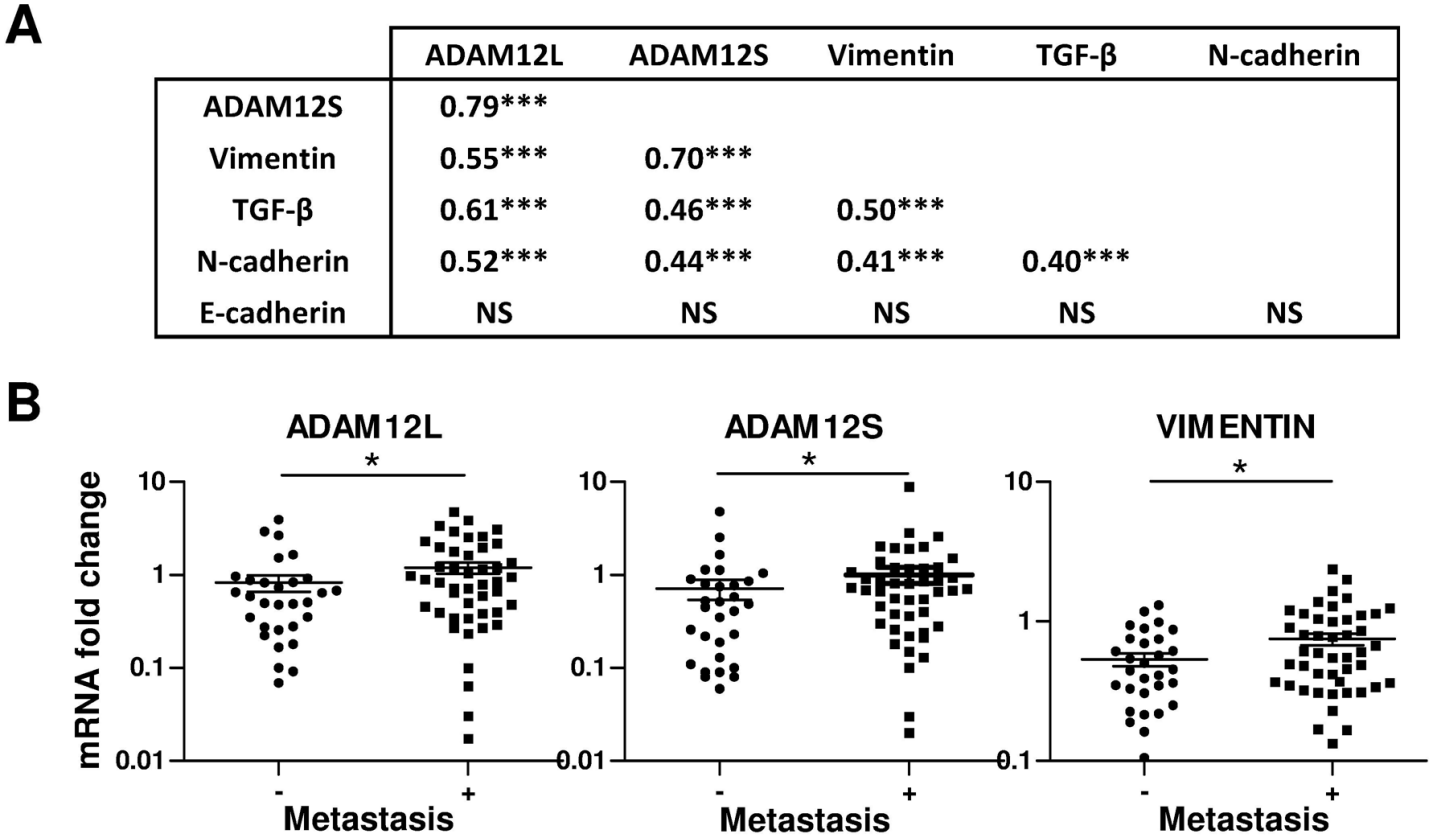
Expression of ADAM12 in human breast cancer biopsies (n = 79). ADAM12 expression is correlated with mesenchymal markers (A) and the presence of metastases (B). Total RNA was extracted from breast tissue samples and the steady-state ADAM12L and ADAM12S, N-cadherin, vimentin, TGF-β and E-cadherin mRNA levels were measured by real-time PCR. (A) Spearman correlation analyses of steady-state ADAM12L, ADAM12S, vimentin, E-cadherin, N-cadherin and TGF-β mRNA levels. (B) Comparative analysis of ADAM12L, ADAM12S and vimentin mRNA levels in breast samples from patients without (-) and with (+) metastases. (*, p<0.05; **,p<0.01 ***,p<0.001).

To investigate the physiological relevance of these observations, we next analyzed the expression of ADAM12 in 79 breast tumor samples. Steady-state ADAM12L and ADAM12S, N-cadherin, vimentin, TGF-β and E-cadherin mRNA levels were measured by real-time PCR. As shown in Fig 2A, expression of ADAM12L and ADAM12S was significantly correlated with that of N-cadherin (R = 0.52 and R = 0.44, p<0.001), vimentin (R = 0.55 and R = 0.70, p<0.001) and TGF-β (R = 0.61 and R = 0.46, p<0.001) but not with that of E-cadherin. As previously demonstrated in liver cancer [6], ADAM12L and ADAM12S mRNA levels were highly correlated (R = 0.79, p<0.001). Importantly, the increased expression of ADAM12L (0.8±0.9 versus 1.2±1.1, p<0.05) and ADAM12S (0.7±0.9 versus 1±1.3, p<0.05) in breast samples was associated with the presence of metastases supporting evidences for the involvement of ADAM12 in human tumor aggressiveness (Fig 2B). Together, these data demonstrated that expression of ADAM12 is correlated with the expression of EMT markers in breast cancers and confirmed the association of ADAM12 expression with aggressiveness.

### ADAM12 Expression Is Induced in Epithelial Cells that Undergo TGF-β-Dependent EMT

**Fig 3:**
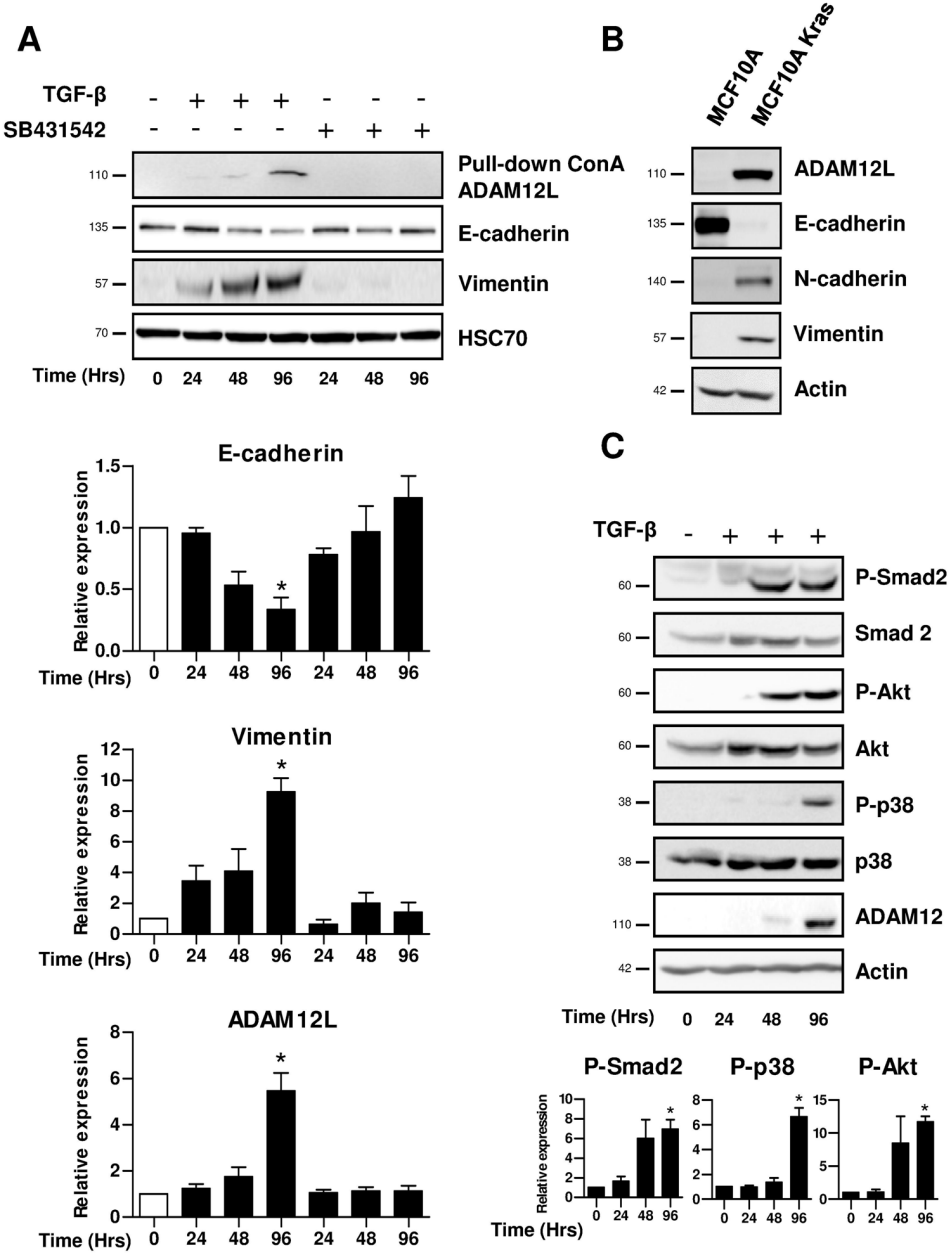
ADAM12 expression is induced in breast epithelial cell line treated with TGF-β. (A), MCF10A cells were stimulated with TGF-β (5ng/ml) for 24, 48 and 96 hours and control cells were incubated with the type I TGF-β receptor inhibitor, SB431542 (10 μM). Concanavalin A-immobilized magnetic nanoparticles were used for selective enrichment of glycoproteins including ADAM12. The levels of vimentin, E-cadherin and ADAM12 were determined by western blot analyses. Top, representative western blots. Bottom, densitometric analyses of protein amounts in cells. Results are expressed as mean±SD from 3 independent experiments (*, p<0.05). (B) Western blot analyses of protein extracts from MCF10A and K-RAS transformed MCF10A epithelial cells cultured for 48h. (C) Cells were stimulated with TGF-β (5ng/ml) for 24, 48 and 96 hours and levels of phosphorylated Ser465/Ser467P-Smad2, Ser473P-Ak and Thr180/Tyr182P-p38 were determined by western blot analysis. Immunoblots for ADAM12 and actin are shown as controls. Top, representative western blots. Bottom, densitometric analyses of protein amounts in cells. Results are expressed as mean±SD from 3 independent experiments (*, p<0.05).

To characterize the dynamics of ADAM12 expression during EMT, we used the non-transformed human breast mammary epithelial cell line MCF10A. As expected, TGF-β treatment rapidly induced the expression of the mesenchymal marker vimentin and reduced the expression of the epithelial marker E-cadherin (Fig 3A). ADAM12 which is not expressed in epithelial cells at the basal state (time 0) is detected at 48 hours and significantly increased 96 hours after the onset of TGF-β stimulation. Western blotting identified expression of a 110kDa band corresponding to the ADAM12L preform, the mature form being undetectable in these conditions. As control, epithelial cells treated with the TβRI inhibitor, SB431542 did not show mesenchymal transition and ADAM12L expression. Using immunofluorescence experiments, we confirmed decrease of E-cadherin, increase of vimentin staining and actin fibers formation after TGFβ treatment (S2 Fig).

To confirm the association of ADAM12L expression with EMT in mammary epithelial MCF10A cells, we showed that Kras-transformed MCF10A cells which display EMT phenotype highly expressed ADAM12L (Fig 3B). Because TGF-β-induced EMT has been associated with changes in TGF-β-signaling pathways [40], we next analyzed Smad-dependent and Smad-independent pathways in our model. As shown in Fig 3C, TGF-β treatment induced both the Smad-dependent pathway characterized by the phosphorylation of Smad2 and the Smad-independent pathways characterized by the phosphorylation of Akt and p38 MAPK. Our results demonstrate that the expression of ADAM12L is induced in non-malignant breast epithelial cells stimulated by TGF-β suggesting that ADAM12L is a new marker for TGF-β-dependent EMT.

### Overexpression of ADAM12L Induces Mesenchymal Phenotype in MCF10A Mammary Epithelial Cells

**Fig 4:**
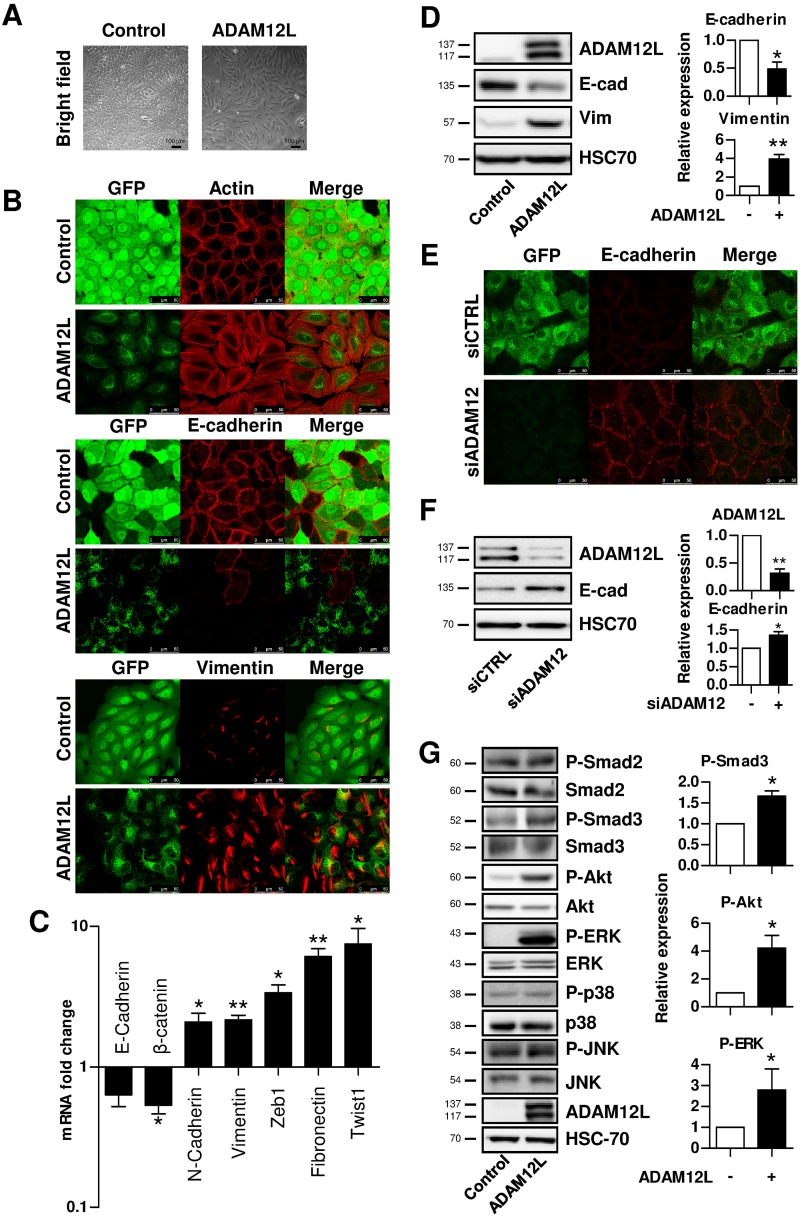
Overexpression of ADAM12L in MCF10A breast epithelial cells induces a mesenchymal phenotype. MCF10A cells were infected with lentiviruses expressing either GFP-ADAM12L fusion protein (ADAM12L) or the control protein GFP (Control). After antibiotic selection, cells were further enriched using flow cytometry and cultured for 48 hours. (A) Bright field pictures were captured to show cell morphology. (B) Cells were fixed and immunostained for E-cadherin, vimentin or stained with rhodamine-conjugated phalloidin to monitor actin stress fibers cytoskeletal actin. (C) Real-time PCR analyses of epithelial (E-cadherin and β-catenin) and mesenchymal (N-cadherin, vimentin, Zeb1, fibronectin and Twist) markers. Results are expressed as fold changes in ADAM12L-overexpressing cells compared with control cells. (D) Western blot analyses of E-cadherin and vimentin in ADAM12L-overexpressing cells. (E and F) ADAM12-overexpressing MCF10A cells were transfected with 100 nM non-targeted siRNA (siControl) or ADAM12 siRNA (siADAM12). After 48 hours, E-cadherin expression was analyzed by Western blotting (E). The efficiency and specificity of RNA interference in cell extracts was confirmed by ADAM12 blots (F). (G) Western blot analyses of phosphorylated Ser465/Ser467P-Smad2, Ser423/Ser425P-Smad3, Ser473P-Akt, Thr202/Tyr204P-ERK, Thr180/Tyr182P-p38 and Thr183/Thr185P-JNK in ADAM12L-overexpressing cells and control cells, 48 hours post-seeding. Immunoblots for ADAM12L and HSC-70 are shown as control. Left, representative western blots. Right, densitometric analyses of protein amounts in cells. Results are expressed as mean±SD from 3 independent experiments (*, p<0.05; **, p<0.01).

To explore the role of ADAM12L in EMT, we exogenously expressed GFP-ADAM12L C-terminal fusion protein in MCF10A cells using a lentiviral expression system. As illustrated in Fig 4A and 4B, enforced expression of ADAM12L in MCF10A cells triggered cellular morphologic changes. ADAM12L-overexpressing MCF10A cells exhibited a spindle shape, fibroblast-like phenotype with accumulation of stress fibers and loss of cell-cell contact assessed by dramatic reduction of E-cadherin and increased vimentin immunostaining. By contrast, MCF10A control cells retained an epithelial phenotype that include rounded-shaped cells, high E-cadherin and low vimentin labeling, cortical staining of actin and cell-cell contacts. To investigate the induction of EMT transcriptional program in ADAM12L-overexpressing MCF10A cells, we performed gene expression analyses. We showed a decrease in mRNA levels of the epithelial markers that include E-cadherin and β-catenin and an increase in mRNA levels of mesenchymal markers that include N-cadherin, vimentin, fibronectin and the transcriptional repressors Zeb1 and Twist (Fig 4C). These results were confirmed by western blotting, showing that E-cadherin is strongly down-regulated whereas vimentin is up-regulated in ADAM12L-overexpressing MCF10A (Fig 4D). Importantly, silencing ADAM12 expression in ADAM12L-overexpressing MCF10A reverse the expression of the epithelial marker, E-cadherin (Fig 4E and 4F).

Because we have previously shown that ADAM12 overexpression modulates Smad-dependent TGF-β and PI3K/Akt pathways [22,24], we investigated the activation of signaling pathways in ADAM12L-overexpressing MCF10A cells. As illustrated in Fig 4G, ADAM12L-overexpressing cells showed significant increase amount of phosphorylated forms of Smad3 (^Ser423/Ser425^P-Smad3), Akt (^Ser473^P-Akt) and ERK (^Thr202/Tyr204^P-ERK), while phosphorylation of Smad2 (^Ser465/Ser467^P-Smad2), p38 (^Thr180/Tyr182^P-p38) and JNK (^Thr183/Thr185^P-JNK) was not significantly modified (not shown). Interestingly the increase in Smad3 phosphorylation was always more pronounced than for Smad2 suggesting that the effect of ADAM12L is more likely Smad3-specific. In accordance with this, Smad3 but not Smad2 has been shown critical to induce TGF-β-mediated EMT [41,42]. Note that the expression of both immature (137 kDa) and activated (117 kDa) GFP-ADAM12L forms was detected in ADAM12L-overexpressing MCF10A cells suggesting that the maturation process is functional in these cells.

### ADAM12L-Induced EMT Is Independent of Its Catalytic Activity but Requires Its Cytoplasmic Tail

**Fig 5:**
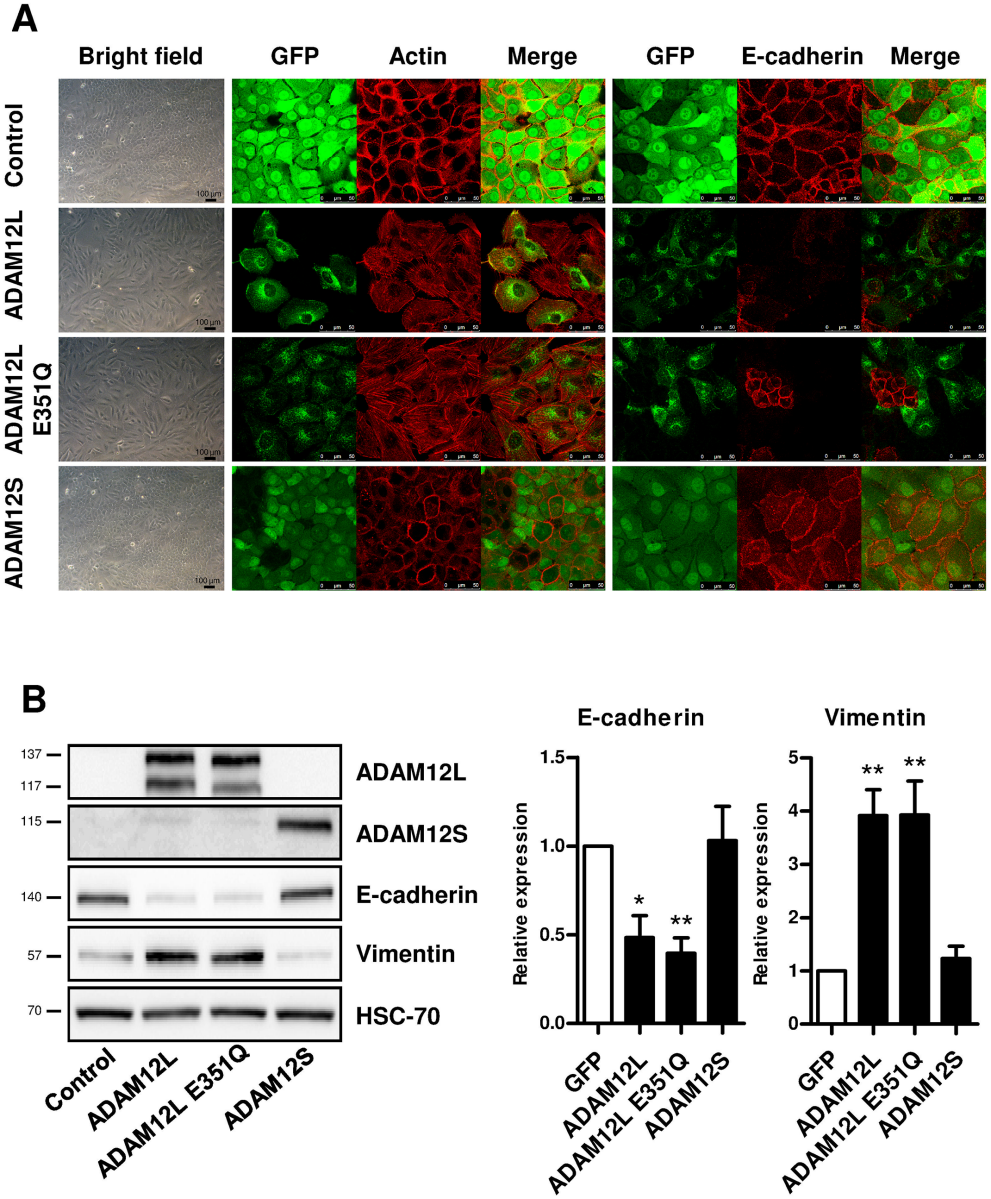
ADAM12-dependent EMT does not require catalytic activity and is specific of ADAM12L. MCF10A cells were infected with lentiviruses expressing either the fusion proteins GFP-ADAM12L, GFP-ADAM12-E351Q, GFP-ADAM12S or the control protein GFP (Control). After antibiotic selection, cells were further enriched using flow cytometry and cultured for 48 hours. (A) Bright field pictures were captured to show cell morphology. Cells were fixed and immunostained for E-cadherin or stained with rhodamine-conjugated phalloidin to monitor actin stress fibers cytoskeletal actin. (B) The levels of vimentin and E-cadherin were determined by western blot analyses. Left, representative western blots. Immunoblots for ADAM12L, ADAM12S and HSC-70 are shown as control. Right, densitometric analysis of protein amounts. Results are expressed as mean±SD from 6 independent experiments (*, p<0.05; **, p<0.01).

**Fig 6:**
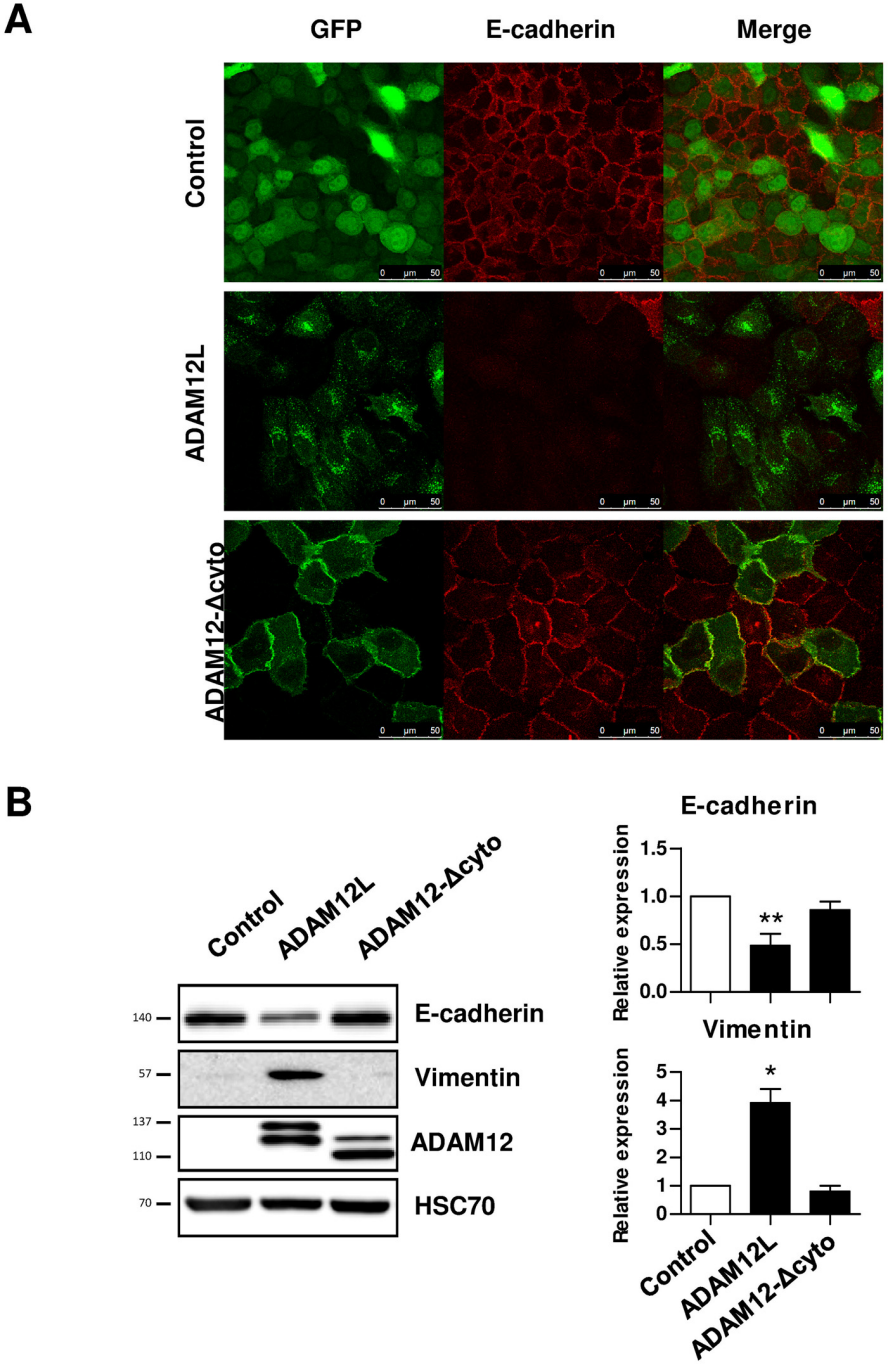
The cytoplasmic tail is required for ADAM12L-induced EMT. MCF10A cells were infected with lentiviruses expressing either the fusion proteins GFP-ADAM12L, the cytoplasmic domain deletion mutant (GFP-ADAM12L-Δcyto) or the control protein GFP (Control). After antibiotic selection, cells were further enriched using flow cytometry and cultured for 48 hours. (A) Cells were fixed and immunostained for E-cadherin (B) The levels of vimentin and E-cadherin were determined by western blot analyses. Left, representative western blots. Immunoblots for ADAM12 and HSC-70 are shown as control. Right, densitometric analysis of protein amounts. Results are expressed as mean±SD from 3 independent experiments (*, p<0.05).

To further explore the molecular mechanisms underlying this novel modulation of EMT by ADAM12L, we next compared MCF10A cells which exogenously expressed either ADAM12L or the catalytically deficient ADAM12-E351Q mutant or the short ADAM12S form. As illustrated in Fig 5A, the catalytically deficient ADAM12-E351Q mutant induced a mesenchymal phenotype to an extent similar to that of wild-type ADAM12L suggesting that proteolytic activity of ADAM12L is not required for EMT induction. In contrast, ADAM12S-overexpressing MCF10A cells retained an epithelial phenotype demonstrating that ADAM12L is specifically involved in EMT. To validate these observations, we next quantified the amount of the epithelial marker E-cadherin and the mesenchymal marker vimentin. In accordance with the mesenchymal phenotype, we found a significant increased expression of vimentin and a decreased expression of E-cadherin in ADAM12L- and ADAM12-E351Q mutant-overexpressing cells (Fig 5B). In contrast, E-cadherin and vimentin expression in ADAM12S-overexpressing cells was similar to that of control cells demonstrating the differential role of the two forms of ADAM12. To further investigate the mechanisms involved in ADAM12L-induced EMT, we generated MCF10A cells overexpressing cytoplasmic domain deletion mutant (ADAM12L-Δcyto). As shown in Fig 6, the cytoplasmic tail is required for the ADAM12L-induced EMT since overexpression of the deletion mutant failed to induce a mesenchymal phenotype but retained high cadherin expression and cell-cell contacts. As previously reported, ADAM12L-Δcyto is targeted to the cell membrane [43].

### Overexpression of ADAM12L Induces Chemoresistance

**Fig 7:**
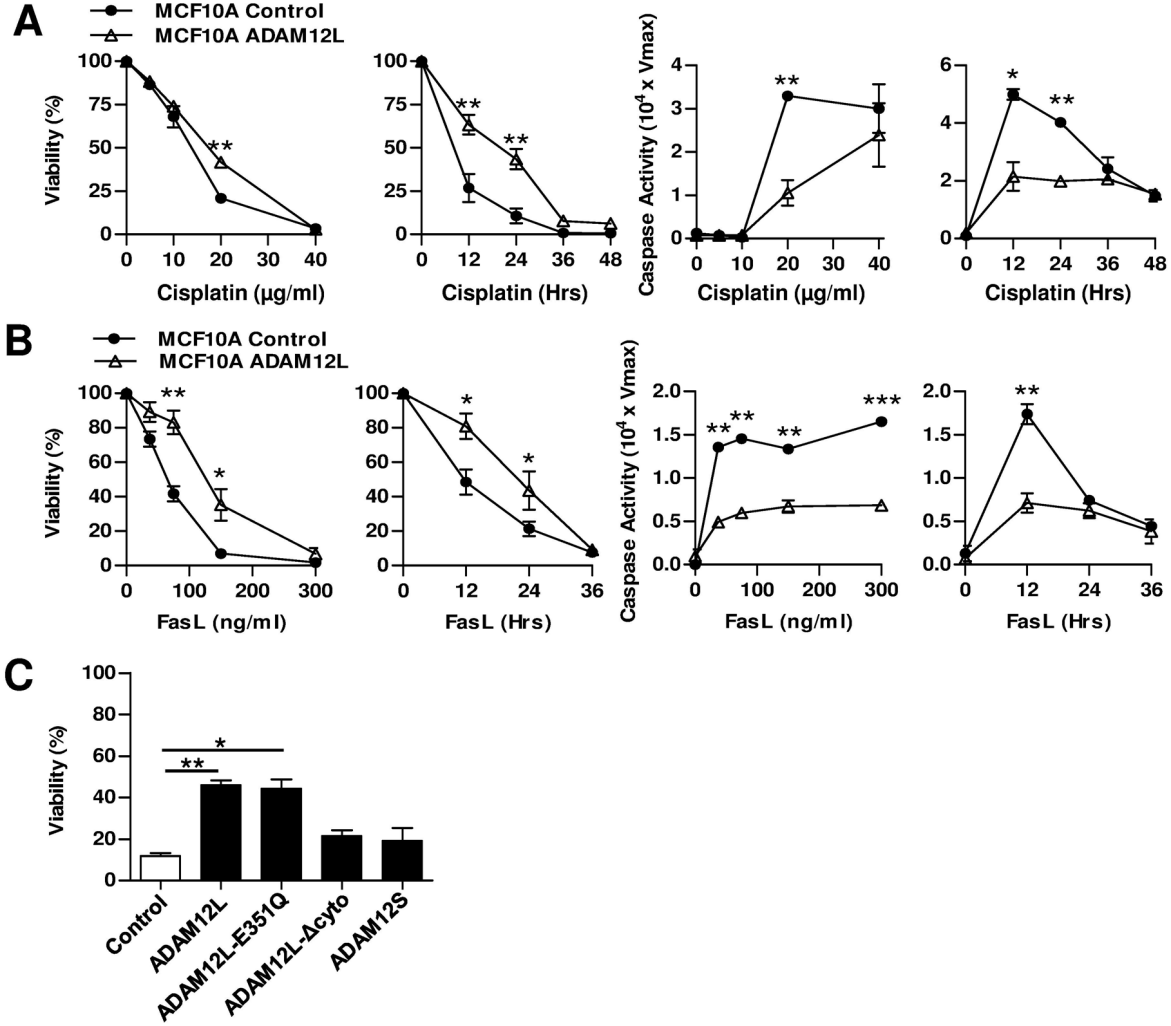
Overexpression of ADAM12L induces chemoresistance in MCF10A cells. (A) Cisplatin-induced apoptosis is analyzed by quantification of cell viability and caspase 3/7 activity. Dose effects are measured after 24h of treatment and kinetic effects are measured with a dose of 20μg/ml. (B) Fas-L induced apoptosis is analyzed by quantification of cell viability and caspase 3/7 activity. Dose effects are measured after 24h of treatment and kinetic effects are measured with a dose of 75 ng/ml. All results are expressed as the mean ±SD from four independent experiments (*, p<0.05; **, p<0.01). (C) Comparative analysis of cell viability between MCF10A cells overexpressing either ADAM12L, ADAM12L-E315Q, ADAM12L-Δcyto or ADAM12S compared with control cells after 24H of cisplatin treatment with a dose of 20μg/ml.

Because EMT phenotype has been associated with chemoresistance, we investigated the effects of cisplatin on ADAM12L-overexpressing cells. As shown in Fig 7A, ADAM12L overexpression confers resistance to cisplatin-induced death. This effect is correlated with a decrease in caspase 3/7 activity. In accordance with these results, we showed that ADAM12L-overexpressing cells are protected against FasL-induced apoptosis supporting the implication of ADAM12L in death resistance (Fig 7B). As expected, ADAM12LΔCyto- and ADAM12S-overexpressing cells did not show significant resistance to cisplatin while ADAM12L-E351Q-overexpressing cells are chemoresistant to a similar extend that of ADAM12L-overexpressing cells (Fig 7C). This effect of ADAM12L is specific of the non-cancerous human breast epithelial cell line MCF10A since overexpression of ADAM12L in MCF7, MDA-MB231 and MDA-MB436 cells did not induce cisplatin chemoresistance (S3A Fig). Accordingly, overexpression of ADAM12L in these breast cancer cell lines did not modify E-cadherin and vimentin expression (S3B Fig).

Together, these results demonstrate that expression of ADAM12L in the non-malignant breast epithelial cell line MCF10A induces a mesenchymal phenotype linked to the acquisition of chemoresistance. Of note, these effects are associated with a decrease in cell proliferation without changes in migration and invasion (S4 Fig).

### ADAM12L Expression Contributes to EMT through TGF-β Receptor-Dependent and Independent Pathways

**Fig 8:**
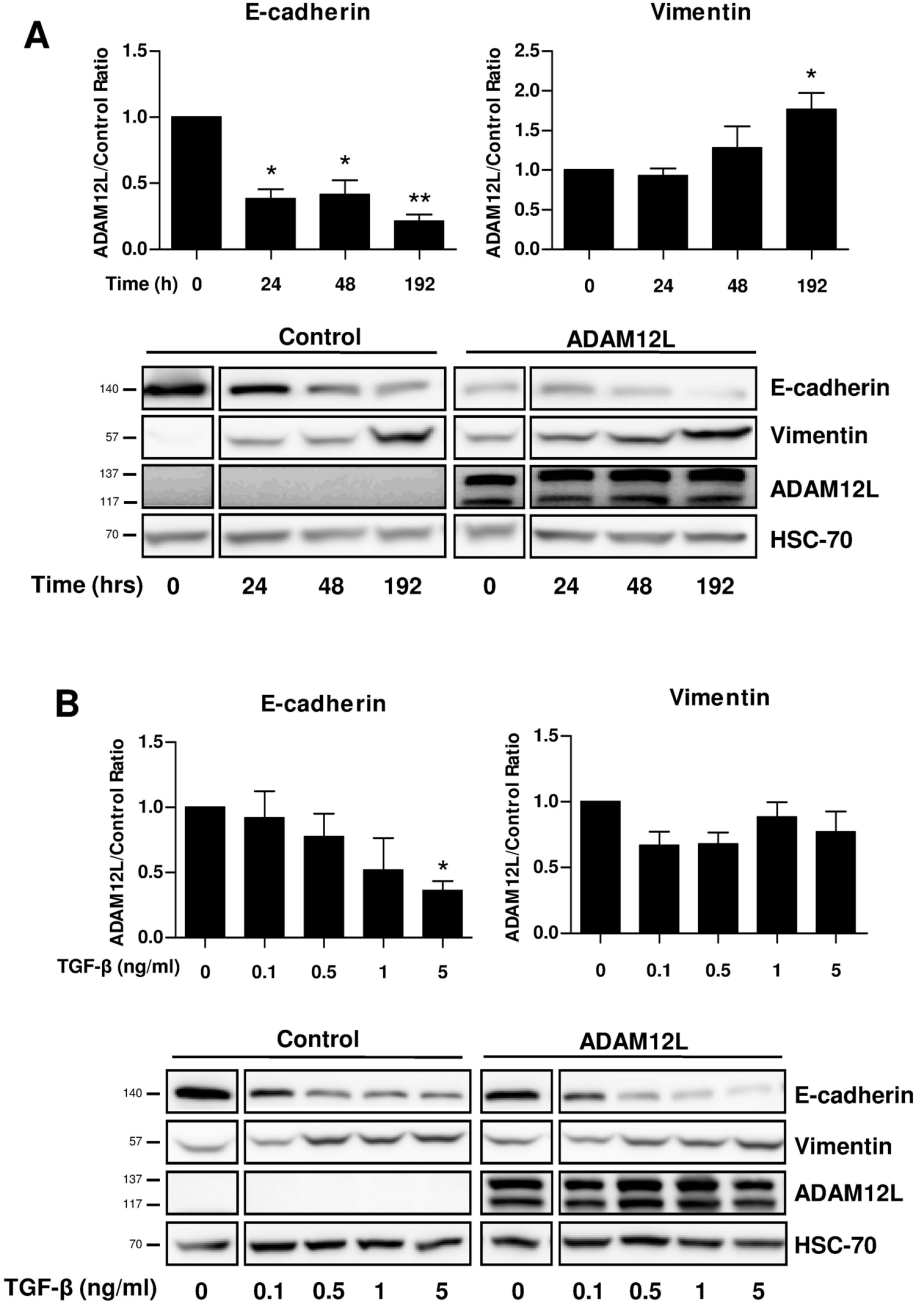
ADAM12L expression facilitates TGF-β-dependent EMT. MCF10A overexpressing GFP-ADAM12L or GFP (control) were treated with TGF-β for indicated period of time (A) and for increasing concentrations of TGF-β (B). Dose effects are measured after 96h of treatment and kinetic effects are measured with a dose of 5ng/ml. The expression of vimentin and E-cadherin were analyzed by western blot and quantified by densitometry. Top, Results are expressed as ratio of protein expression in GFP-ADAM12L-overexpressing cells to control cells (mean±SD) from four independent experiments (*, p<0.05; **, p<0.01). Bottom, representative western blots.

Because ADAM12L expression is induced during TGF-β-induced EMT (Fig 3) and because ADAM12L overexpression is sufficient to induce EMT (Fig 4), we next investigated the overlaps between TGF-β and ADAM12L effects in promoting EMT. For that purpose, we compared the effect of TGF-β treatment of ADAM12L-overexpressing MCF10A cells with that of control cells. As illustrated in Fig 8A, we observed a stronger decrease in E-cadherin and a higher increase in vimentin expression in ADAM12L-overexpressing cells compared to control cells. This effect is sensitive to the concentration of TGF-β for E-cadherin expression while the amount of vimentin between ADAM12L-overexpressing cells and controls differed only for weak TGF-β concentration since TGF-β treatment rapidly saturated the induction of vimentin (Fig 8B). These data demonstrate that the presence of ADAM12L in epithelial MCF10A cells enhances the effect of TGF-β in inducing EMT phenotype. Note that blocking TGF-β-dependent induction of endogenous ADAM12L expression in MCF10A slightly but not significantly prevent the E-cadherin decrease and vimentin increase upon TGF-β stimulation (S5A and S5B Fig). In addition, inhibiting ADAM12 expression in MDA-MB231 and MDA-MB436 mesenchymal cells that express endogenous ADAM12 did not reverse their mesenchymal phenotype (S5C Fig). Together these data suggest that ADAM12L contributes but is not essential to TGF-β-induced EMT in cultured MCF10A cells.

**Fig 9:**
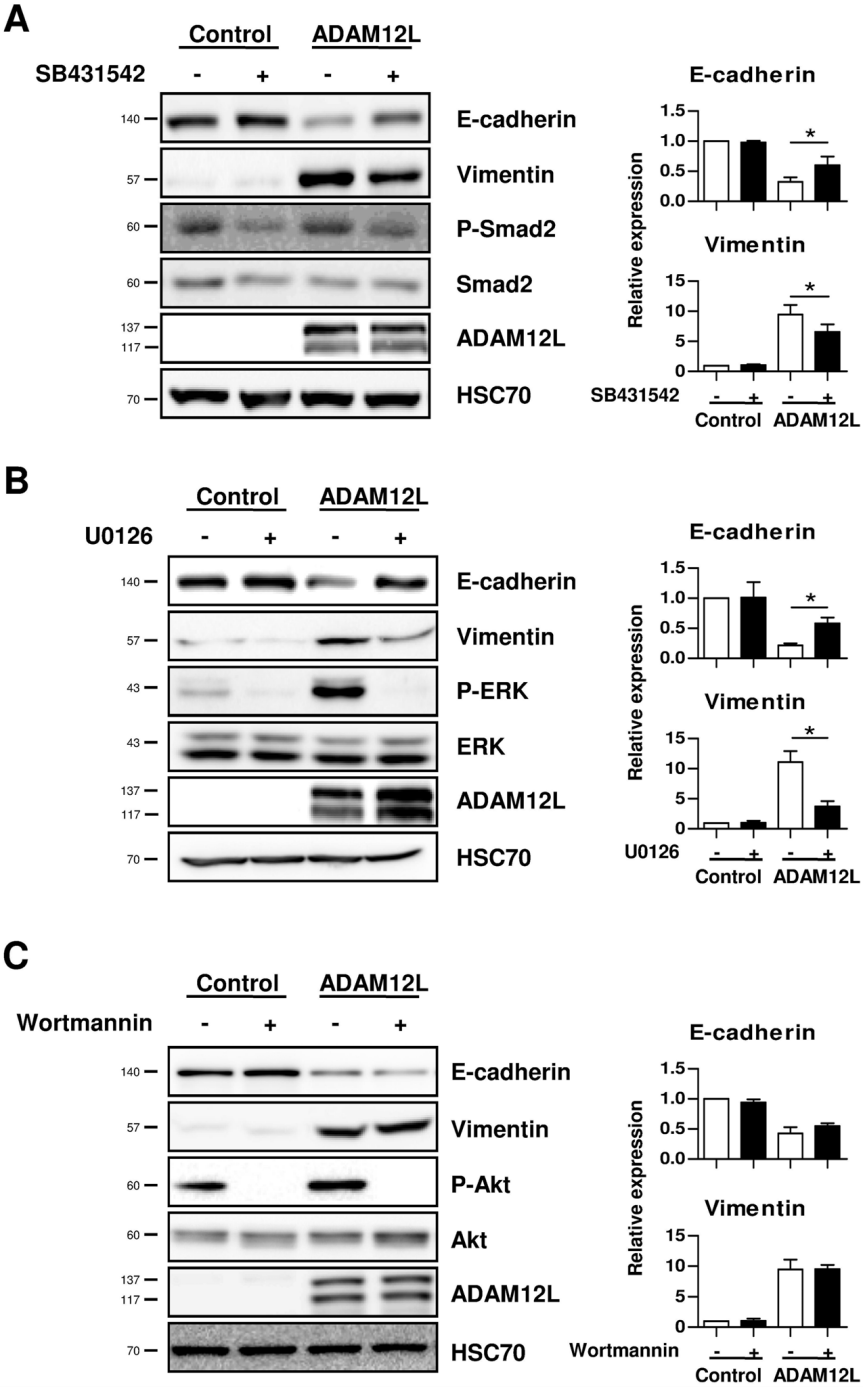
Inhibition of TβRI or ERK-MAPK reverses ADAM12L-induced mesenchymal phenotype. MCF10A cells overexpressing GFP-ADAM12 were treated (+) or not (-) with (A) the selective inhibitor of TGFβRI, SB431542 (10μM), (B) a highly selective inhibitor of both MEK1 and MEK2, U0126 (10μM), and (C) the PI3K inhibitor, Wortmannin (10μM) for 72 hours. The expression of vimentin and E-cadherin was analyzed by western blot and quantified by densitometry. Data are expressed as mean±SD from four independent experiments (*, p<0.05; **, p<0.01).

In order to further evaluate the overlap between ADAM12L and TGF-β effects, we investigated whether the inhibition of TGF-β signaling decreased EMT phenotype induced by ADAM12L. For that purpose, cells were treated with a selective inhibitor of TGFβR1 (SB431542) that blocks the initialization of TGF-β dependent signaling pathways at the receptor level. As shown in Fig 9A, SB431542 treatment of ADAM12L-overexpressing cells induced a significant increase in E-cadherin expression (2 ± 0.2 fold, p<0.05) and a slight decrease in vimentin expression (0.74 ± 0.1 fold, p<0.05) suggesting that inhibition of TGF-β receptors partially restored the epithelial phenotype. The efficiency of SB431542 treatment was shown by the decrease in Smad2 phosphorylation. Because expression of ADAM12L is associated with changes of Smad-independent signaling pathways (Fig 4G), we investigated the effects of ERK and PI3K inhibitors on ADAM12L-induced mesenchymal phenotype. ADAM12L-overexpressing MCF10A cells were incubated with either a highly selective inhibitor of MEK/ERK kinases, U0126 or the specific inhibitor of phosphoinositide 3-kinase (PI3K), Wortmannin. As shown in Fig 9B and S6 Fig, U0126 treatment induced a dramatic decrease in vimentin and N-cadherin and an increase in E-cadherin. Unlike U0126, treatments with Wortmannin did not reverse EMT phenotype in ADAM12L-overexpressing cells (Fig 9C and S6 Fig). Together, these results provide strong evidence for the implication of TGFβR and MEK/ERK-dependent pathways in ADAM12L-induced mesenchymal phenotype.

## Discussion

Epithelial mesenchymal transition is a complex cell-biological program whereby epithelial tumor cells loose polarity and cell-cell adhesion to acquire mesenchymal phenotype. Using transcriptomic data from a large panel of breast cancer cell lines, we demonstrated that ADAM12 clustered with EMT gene signature suggesting that ADAM12 is a component of EMT protein networks. We further showed that expression of ADAM12 is correlated with expression of EMT markers in human breast cancer samples and the presence of metastases. The association of ADAM12 with breast cancer aggressiveness is in accordance with the induction of metastasis *in vivo* by ADAM12-overexpressing breast cell lines [3,15] and the association of ADAM12 with metastases in triple-negative breast cancer [44]. Additionally ADAM12 expression has been recently identified in transcriptomic data from claudin-low tumors [45] which have been previously associated with EMT core signature [46]. Together these studies point out ADAM12 as a new EMT marker.

### ADAM12 Is Associated with TGF-β Dependent EMT

ADAM12 has been identified as a regulator of mesenchymal cell differentiation while its expression has been mainly associated with cancer epithelial cells suggesting that increased ADAM12 expression in tumor cells is associated with dedifferentiation of epithelial cells towards mesenchymal phenotype. This point was clearly demonstrated by our results showing that treatment of the non-malignant breast epithelial cell line MCF10A with TGF-β induced EMT and ADAM12 expression. In support of this, ADAM12 expression has been identified as a gene primarily induced in MCF10A cells silenced for TIF1γ, a negative regulator of TGF-β transcriptional activity, and characterized by EMT phenotype [47]. Similarly, down-regulation of SnoN, a negative regulator of TGF-β signaling, has been shown to enhance both EMT in lung and breast cancer cells [48] and TGF-β-induced expression of ADAM12 [49]. Interestingly, there is a direct relationship between ADAM12 expression and cell differentiation processes regulated by TGF-β. On one hand, TGF-β inhibits mesenchymal cell differentiation promoting progenitor cells proliferation and ADAM12 is mainly associated with undifferentiated proliferative state of mesenchymal cells such as chondrocytes [50], adipocytes [51], myoblasts [52] and osteoblasts [53]. On the other hand, TGF-β acts as a major inducer of EMT and we now demonstrate that TGF-β-induced ADAM12L expression in epithelial cells is correlated with EMT process. Of note, EMT is associated with the gain of epithelial stem-cell properties [54] and recent findings demonstrated functional association of ADAM12 with TGF-β-dependent stem cell differentiation [55] and identified ADAM12 as a marker of embryonic stem cells [56] thereby suggesting that ADAM12 might be an important player in stem cell differentiation.

### The Long but Not the Short ADAM12 Form Induces Mesenchymal Phenotype

A major finding in the present study is the demonstration that ADAM12L expression in non-malignant human breast epithelial cell line MCF10A induces phenotypical changes associated with EMT that include loss of cell-cell contact, accumulation of stress fibers and increased expression of specific markers. Importantly we further demonstrate that ADAM12L expression in epithelial cells confers chemoresistance, a known feature of mesenchymal cells. Unlike ADAM12L, the overexpression of ADAM12S does not induce changes in epithelial phenotype. These data are in accordance with the differences previously reported between the long and short forms. ADAM12L but not ADAM12S transcripts were found overexpressed in human non-small-cell lung carcinomas [11] and inversely associated with survival rate in lung adenocarcinoma patients [57]. In human breast cancer, ADAM12L expression has been found higher in early-stage of tumorigenesis, both isoforms being induced in late-stage diseases [15]. Similarly, we show that expression of ADAM12L but not ADAM12S is induced within 48 hours in MCF10A stimulated by TGF-β supporting the hypothesis that ADAM12L is an earlier marker of tumor development. However, ADAM12L over-expression does not affect cell motility and does not induced proliferation of normal epithelial cell lines. Absence of changes in motility have been similarly reported in MCF7 breast cell line [15] and lung cancer cell lines [58] and no proliferation was reported in normal breast epithelial [59]. By contrast, ADAM12L overexpression has been shown to promote proliferation of bronchial epithelial immortalized cells [60] and small cell lung carcinoma cell lines [58] suggesting that the effect of ADAM12L depends on the differentiation state of cells.

### Cooperative Effect of ADAM12L and TGF-β Signaling Pathways in EMT

The functional mechanism associating ADAM12 and TGF-β signaling is supported by our previous work showing that ADAM12 interacts with TβRII which recruits TβRI to induce TGF-β signaling pathways [24]. Numerous ADAM12 interacting proteins are required for TGF-β signaling-dependent EMT such as ILK, ITGB1, PIK3R1, Grb2, and Src. Here, we demonstrate for the first time that ADAM12L expression in MCF10A cells results in both phosphorylation of Smad3 and ERK, two key proteins involved in TGF-β-mediated EMT [42][61]. Together our data support evidence for the involvement of ADAM12 in activation of pathways similar to that induced by TGF-β. In accordance with this hypothesis, we demonstrate that specific inhibitors of TβR1 (SB431542) and MEK/ERK pathway (U0126) can reverse ADAM12L-induced EMT suggesting that ADAM12L acts through the TGF-β receptor and involves ERK pathway in absence of TGF-β ligand. An interesting point is that the effects of ADAM12L and TGF-β ligand are additive thereby providing evidences for a cooperative role of ADAM12L during the course of TGF-β-induced EMT. Because ADAM12 expression is induced by TGF-β [6][62] and that activation of signaling pathways occurs independently of TGF-β stimulation in ADAM12L-overexpressing cells, ADAM12L could induce ligand-independent activation of TGF-β receptors-dependent pathways. Similarly, changes of ADAM12-overexpressing cells toward mesenchymal-like phenotype could be associated with secretion of new potential ligands for TGF-β receptors. In support of this hypothesis, type I collagen which is produced by numerous mesenchymal cells and which regulates ADAM12 translocation to the membrane [26] has been shown to activates EMT through Smad3-dependent pathways in absence of TGF-β stimulation [63].

To conclude, our present study establishes ADAM12 as a new EMT marker in human breast cancers. Importantly, expression of ADAM12L in non-malignant breast epithelial cells promotes EMT through TGF-β receptor-dependent pathways involving ERK phosphorylation. These findings support new functions for ADAM12L in cancer acting as a signaling molecule that contributes to the complexity of TGF-β signaling pathways in EMT process.

## Supporting Information

S1 Fig ADAM12 expression is induced in TGF-β treated immortalized HMEC. Primary human mammary epithelial cells (HMEC) infected with a retrovirus carrying hTERT, H-Ras-V12 Ras and SV40 large T antigen were kindly provided by Dr RA Weinberg (Cambridge, MA, USA) (37). HMECs immortalized by hTERT and Ras were designated HMEC-TR. HMECs immortalized by SV40 large T, hTERT and Ras were designated HMEC-LTR. Cells were treated with 10ng/ml recombinant TGF-β for 15 days. Total RNA was extracted and the steady-state ADAM12L and ADAM12S, N-cadherin, vimentin, TGF-β were measured by real-time PCR.
(TIF)

S2 Fig TGF-β induces EMT in MCF10A cells. MCF10A cells were treated with TGF-β and fixed at indicated times. Cells were immunostained for E-cadherin, vimentin or stained with rhodamine-conjugated phalloidin to monitor actin stress fibers cytoskeletal actin.
(TIF)

S3 Fig Overexpression of ADAM12L in tumoral mammary cell lines did not modify chemoresistance. (A) Cisplatin-induced apoptosis is analyzed by quantification of cell viability and caspase 3/7 activity. Dose effects are measured after 24h of treatment and kinetic effects are measured with a dose of 20μg/ml. Dose effects are measured after 24h of treatment and kinetic effects are measured with a dose of 20μg/ml. All results are expressed as the mean ±SD from four independent experiments (*, p<0.05; **, p<0.01). (B) Western blot analyses of E-cadherin and vimentin in ADAM12L overexpressing tumor cell lines.
(TIF)

S4 Fig Overexpression of ADAM12L does not affect cell migration and reduces cell proliferation. (A) Control MCF10A or ADAM12L-overexpressing MCF10A cells were subjected to a wound healing assay in presence of mitomycin (2.5 μg/ml). The pictures were taken immediately after incision (0 hour) and at 20 hours after incision using a 10× objective. The area of wound was quantified using Java's image J software. Left, representative pictures. Right, quantification of data from four independent experiments. (B) Migration assays in Boyden chambers. Left, representative pictures. Right, quantification of data from four independent experiments. (C) Soft agarose colony formation assays. Left, representative pictures. Right, quantification of data from four independent experiments. (D) Proliferation assays. Control MCF10A or ADAM12L-overexpressing MCF10A cells were subjected to MTT assay at 0, 24, 48 and 72h and doubling time was calculated. Results are expressed as the mean ±SD from four independent experiments (**, p<0.01).
(TIF)

S5 Fig ADAM12 expression is not essential for TGF-β-induced EMT. (A) Validation of effects of Lentiviral shADAM12 Transduction Particles (1, 2 and 3) in ADAM12-overexpressing MCF10A clones (left panel, RT-qPCR). (B) MCF10A clones expressing sh directed against ADAM12 (shADAM12 (1), shADAM12 (2), shADAM12 (3) or control sh (shC)) were treated with TGF-β for 96 hours. E-cadherin and vimentin expression was analyzed by western blots and the amount of proteins was quantified by densitometry. Results are expressed as the mean ±SD of three independent experiments. (C) Stable and transient transfection of MDA-MB-231 and MDA-MB-436 cells with sh and siRNA targeting ADAM12, respectively. Expression of vimentin and E-cadherin was analyzed 48h after seeding using western blots.
(TIF)

S6 Fig Inhibition of TβRI or ERK-MAPK reverses ADAM12L-induced mesenchymal phenotype. MCF10A cells overexpressing GFP-ADAM12 were treated or not with the selective inhibitor of TGFβRI, SB431542 (10μM), a highly selective inhibitor of both MEK1 and MEK2, U0126 (10μM), and the PI3K inhibitor, Wortmannin (10μM) for 72 hours. Cells were fixed and immunostained for E-cadherin.
(TIF)

S1 Table Description of breast cancer cell lines. ER, Estrogen receptor, PR, progesterone receptor, and Her2, human epidermal growth factor receptor 2. (XLSX)

S2 Table Common list of genes upregulated in Basal B cell lines compared with Basal A and Luminal cell lines from Kao et al, 2009 and Neve et al, 2006. (XLS)
