# Supplementary figures and images for: The Disintegrin and Metalloprotease ADAM12 Is Associated with TGF-β-Induced Epithelial to Mesenchymal Transition
Source: PLoS One. 2015 Sep 25;10(9):e0139179. doi: 10.1371/journal.pone.0139179 (PMC4583281; doi:10.1371/journal.pone.0139179)

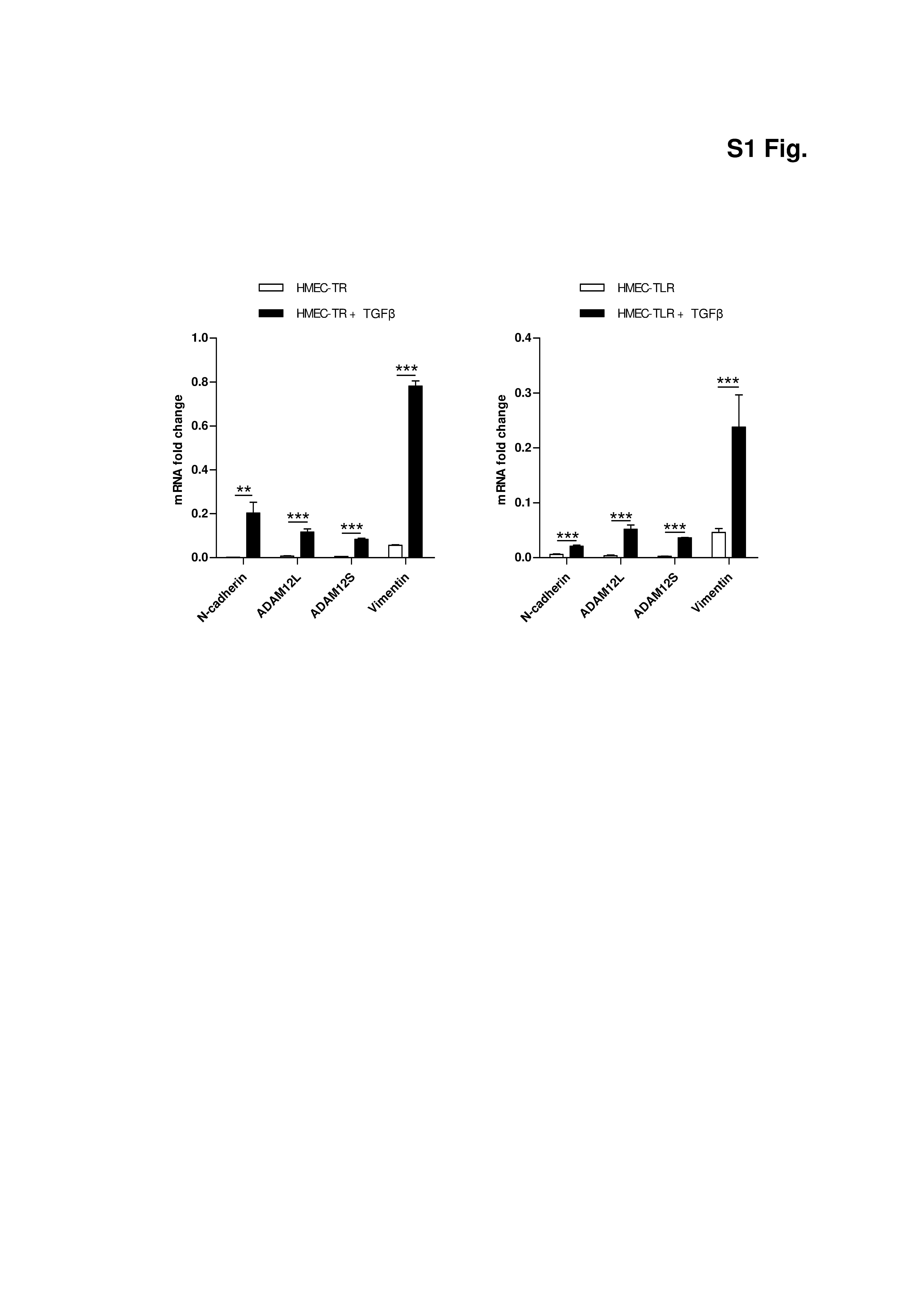

Supplement: S1 Fig — Primary human mammary epithelial cells (HMEC) infected with a retrovirus carrying hTERT, H-Ras-V12 Ras and SV40 large T antigen were kindly provided by Dr RA Weinberg (Cambridge, MA, USA) (37). HMECs immortalized by hTERT and Ras were designated HMEC-TR. HMECs immortalized by SV40 large T, hTERT and Ras were designated HMEC-LTR. Cells were treated with 10ng/ml recombinant TGF-β for 15 days. Total RNA was extracted and the steady-state ADAM12L and ADAM12S, N-cadherin, vimentin, TGF-β were measured by real-time PCR. (TIF) [file pone.0139179.s001.tif]

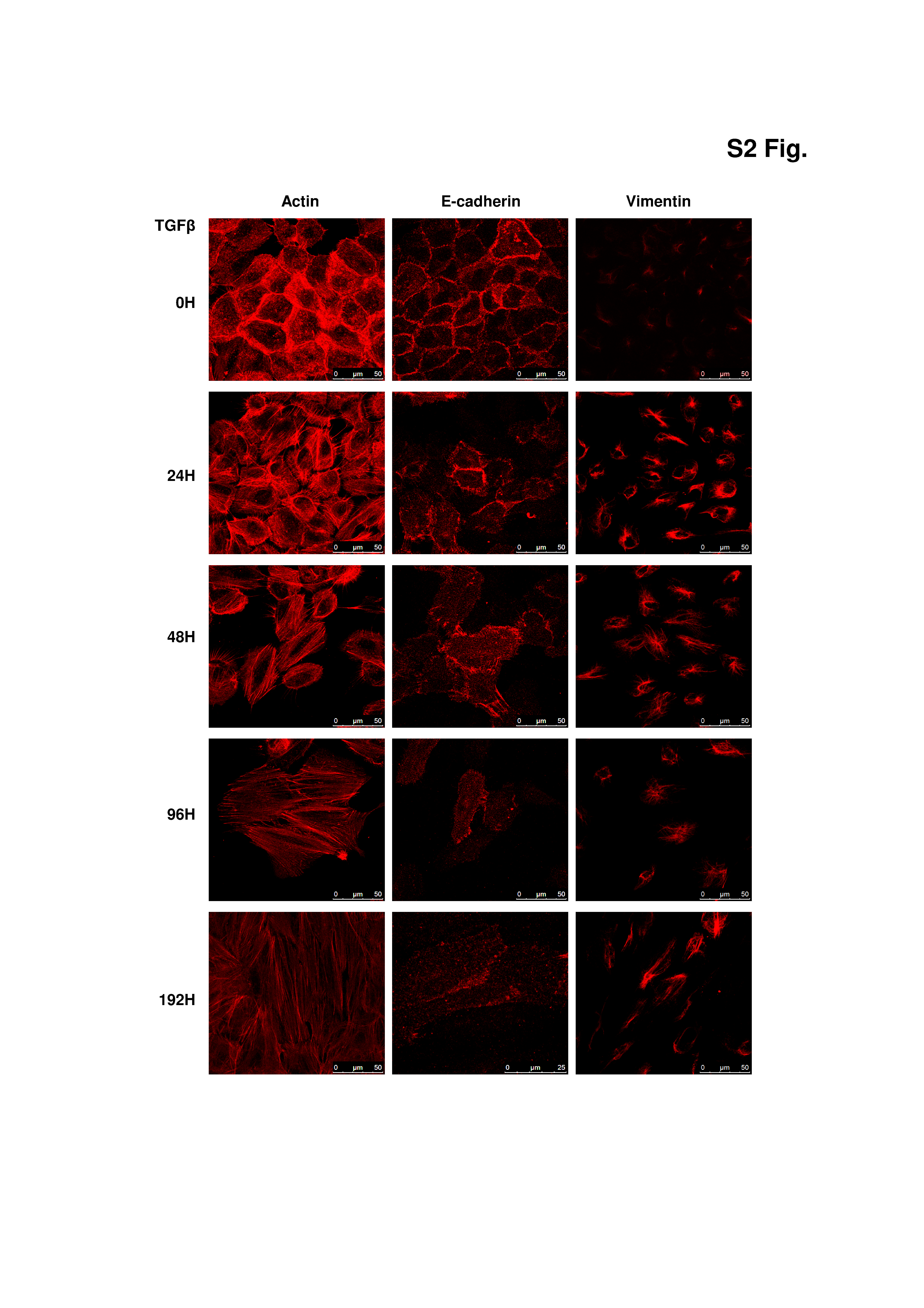

Supplement: S2 Fig — MCF10A cells were treated with TGF-β and fixed at indicated times. Cells were immunostained for E-cadherin, vimentin or stained with rhodamine-conjugated phalloidin to monitor actin stress fibers cytoskeletal actin. (TIF) [file pone.0139179.s002.tif]

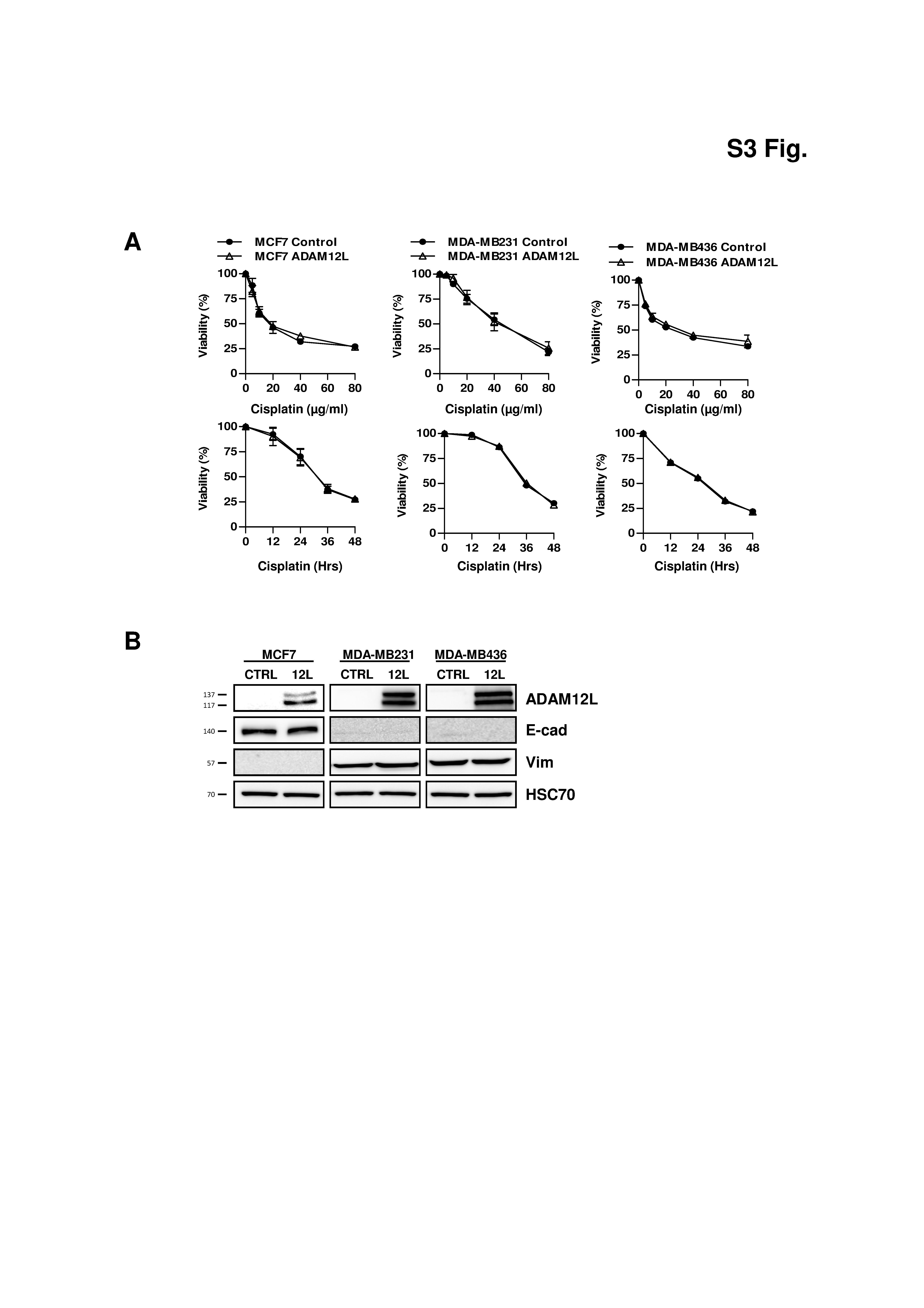

Supplement: S3 Fig — (A) Cisplatin-induced apoptosis is analyzed by quantification of cell viability and caspase 3/7 activity. Dose effects are measured after 24h of treatment and kinetic effects are measured with a dose of 20μg/ml. Dose effects are measured after 24h of treatment and kinetic effects are measured with a dose of 20μg/ml. All results are expressed as the mean ±SD from four independent experiments (*, p<0.05; **, p<0.01). (B) Western blot analyses of E-cadherin and vimentin in ADAM12L overexpressing tumor cell lines. (TIF) [file pone.0139179.s003.tif]

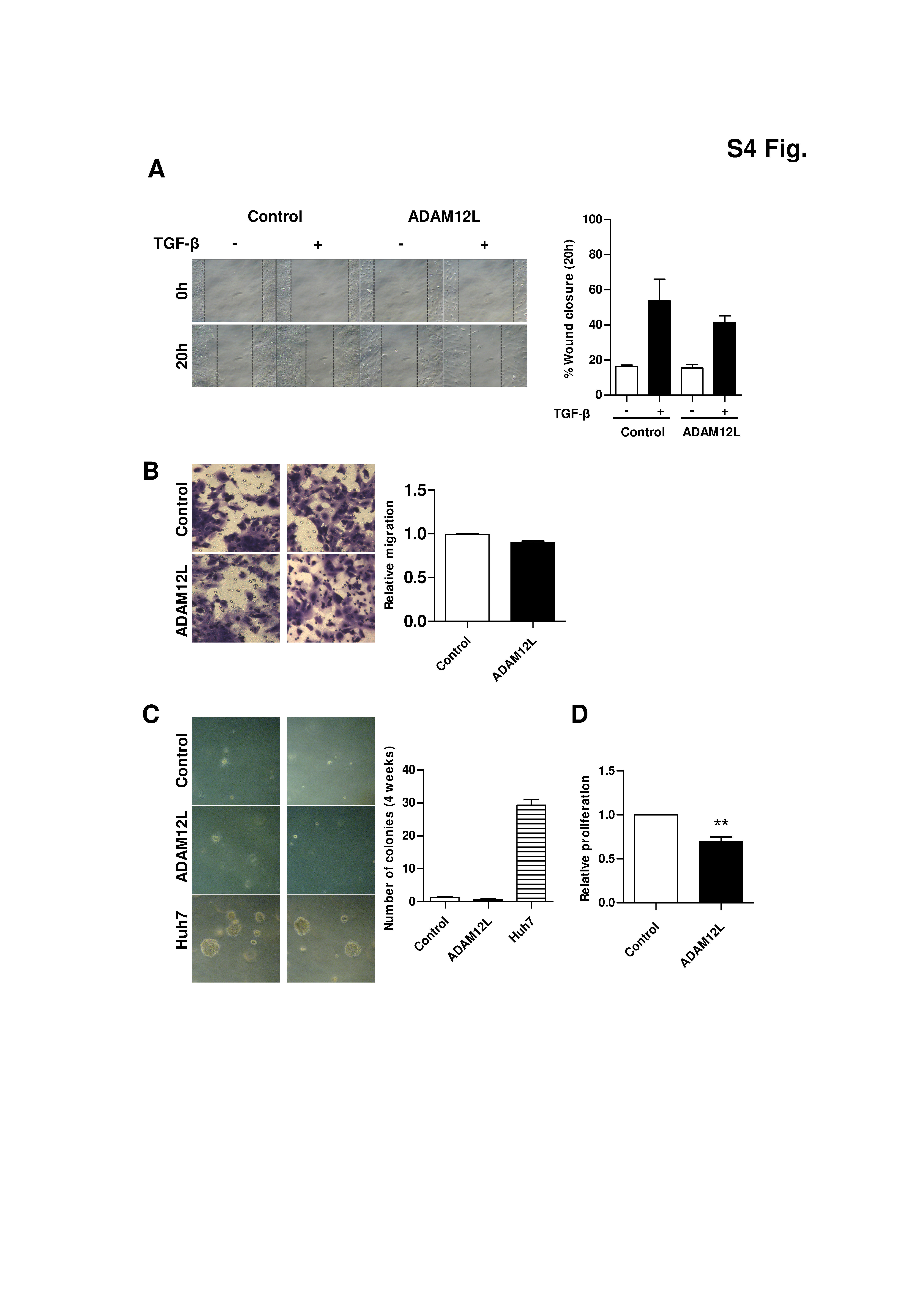

Supplement: S4 Fig — (A) Control MCF10A or ADAM12L-overexpressing MCF10A cells were subjected to a wound healing assay in presence of mitomycin (2.5 μg/ml). The pictures were taken immediately after incision (0 hour) and at 20 hours after incision using a 10× objective. The area of wound was quantified using Java's image J software. Left, representative pictures. Right, quantification of data from four independent experiments. (B) Migration assays in Boyden chambers. Left, representative pictures. Right, quantification of data from four independent experiments. (C) Soft agarose colony formation assays. Left, representative pictures. Right, quantification of data from four independent experiments. (D) Proliferation assays. Control MCF10A or ADAM12L-overexpressing MCF10A cells were subjected to MTT assay at 0, 24, 48 and 72h and doubling time was calculated. Results are expressed as the mean ±SD from four independent experiments (**, p<0.01). (TIF) [file pone.0139179.s004.tif]

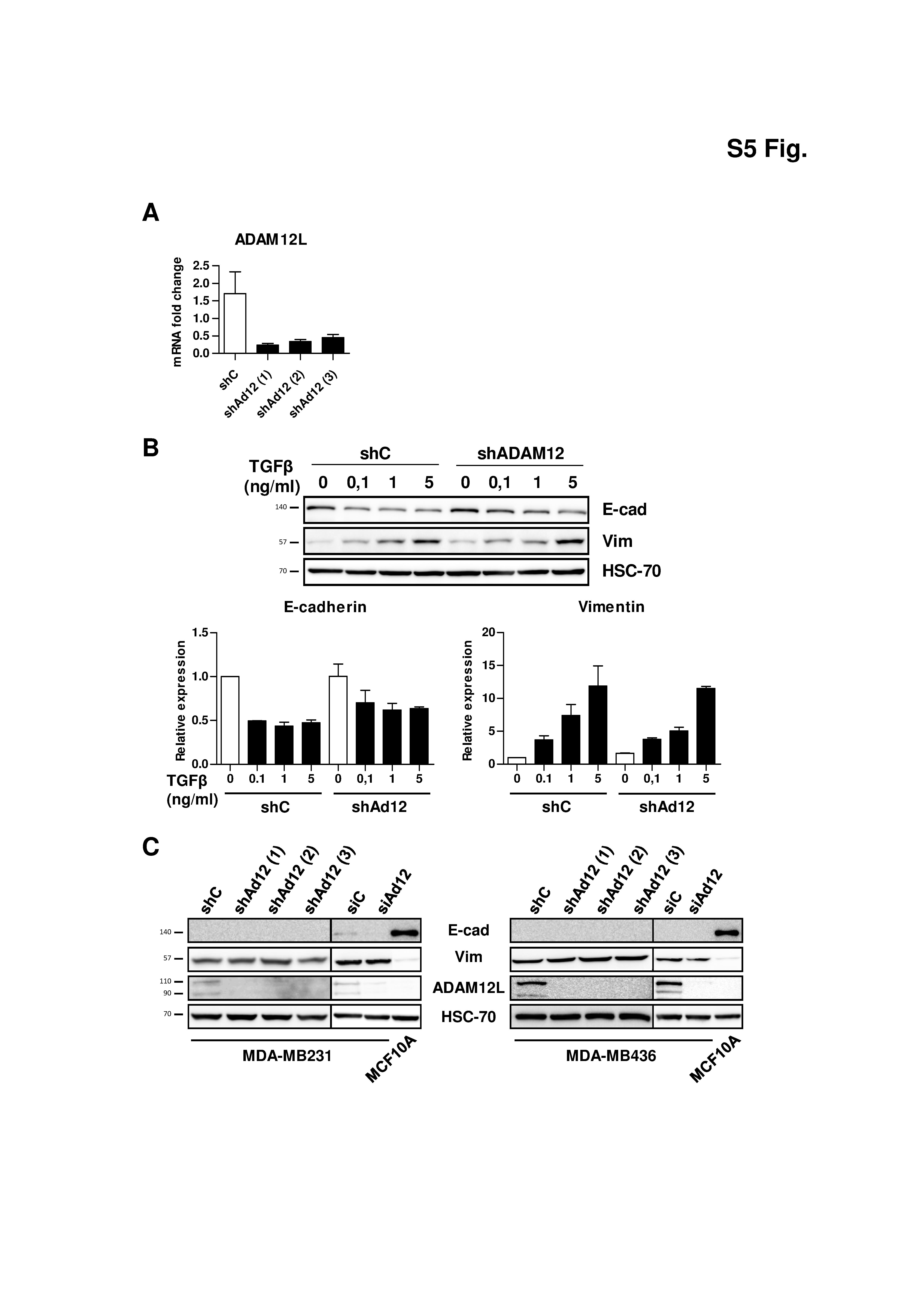

Supplement: S5 Fig — (A) Validation of effects of Lentiviral shADAM12 Transduction Particles (1, 2 and 3) in ADAM12-overexpressing MCF10A clones (left panel, RT-qPCR). (B) MCF10A clones expressing sh directed against ADAM12 (shADAM12 (1), shADAM12 (2), shADAM12 (3) or control sh (shC)) were treated with TGF-β for 96 hours. E-cadherin and vimentin expression was analyzed by western blots and the amount of proteins was quantified by densitometry. Results are expressed as the mean ±SD of three independent experiments. (C) Stable and transient transfection of MDA-MB-231 and MDA-MB-436 cells with sh and siRNA targeting ADAM12, respectively. Expression of vimentin and E-cadherin was analyzed 48h after seeding using western blots. (TIF) [file pone.0139179.s005.tif]

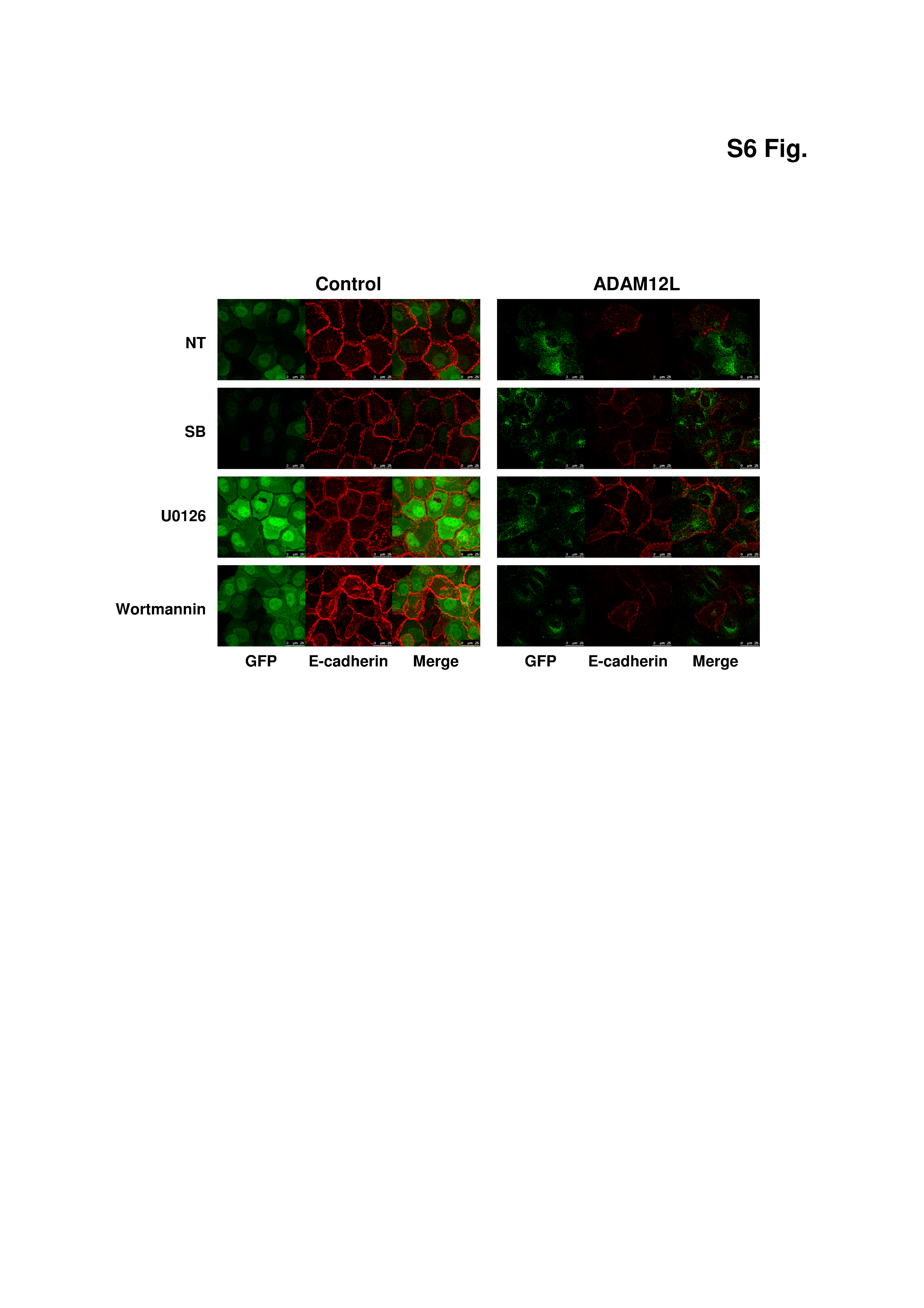

Supplement: S6 Fig — MCF10A cells overexpressing GFP-ADAM12 were treated or not with the selective inhibitor of TGFβRI, SB431542 (10μM), a highly selective inhibitor of both MEK1 and MEK2, U0126 (10μM), and the PI3K inhibitor, Wortmannin (10μM) for 72 hours. Cells were fixed and immunostained for E-cadherin. (TIF) [file pone.0139179.s006.tif]
